# Author Correction: Multi-target mode of action of silver against *Staphylococcus aureus* endows it with capability to combat antibiotic resistance

**DOI:** 10.1038/s41467-021-24538-2

**Published:** 2021-07-01

**Authors:** Haibo Wang, Minji Wang, Xiaohan Xu, Peng Gao, Zeling Xu, Qi Zhang, Hongyan Li, Aixin Yan, Richard Yi-Tsun Kao, Hongzhe Sun

**Affiliations:** 1 Department of Chemistry, State Key Laboratory of Synthetic Chemistry, CAS-HKU Joint Laboratory of Metallomics on Health and Environment, The University of Hong Kong, Hong Kong S.A.R., People’s Republic of China; 2 School of Biological Sciences, The University of Hong Kong, Hong Kong S.A.R., People’s Republic of China; 3 School of Chemistry and Molecular Engineering, East China Normal University, Shanghai, People’s Republic of China; 4 Department of Microbiology, The University of Hong Kong, Hong Kong S.A.R., People’s Republic of China

**Keywords:** Antimicrobials, Antimicrobial resistance, X-ray crystallography, Metals, Proteomics

Correction to: *Nature Communications* 10.1038/s41467-021-23659-y, published online 07 June 2021.

The original version of this Article contained an error in the author affiliations. Affiliation 1 incorrectly read “Department of Chemistry, The University of Hong Kong, Hong Kong S.A.R., People’s Republic of China”. This has now been corrected to “Department of Chemistry, State Key Laboratory of Synthetic Chemistry, CAS-HKU Joint Laboratory of Metallomics on Health and Environment, The University of Hong Kong, Hong Kong S.A.R., People’s Republic of China”. in both the PDF and HTML versions of the Article.

