# Supplementary material for: Multi-target mode of action of silver against Staphylococcus aureus endows it with capability to combat antibiotic resistance
Source: Nat Commun. 2021 Jun 7;12:3331. doi: 10.1038/s41467-021-23659-y (PMC8184742; doi:10.1038/s41467-021-23659-y)
Supplement: Supplementary file 1 — Supplementary information. [file 41467_2021_23659_MOESM1_ESM.pdf]

SUPPLEMENTARY INFORMATION

Multi-target mode of action of silver against *Staphylococcus aureus* endows it with capability to combat antibiotic resistance

Haibo Wang[1], Minji Wang[2,3], Xiaohan Xu[1], Peng Gao[4], Zeling Xu[2], Qi Zhang[1], Hongyan Li[1], Aixin Yan[2], Richard Yi-Tsun Kao[4], & Hongzhe Sun[1*]

[1]*Department of Chemistry, The University of Hong Kong, Pokfulam Road, Hong Kong S.A.R., P. R. China*

[2]*School of Biological Sciences, The University of Hong Kong, Pokfulam Road, Hong Kong S.A.R., P. R. China*

[3]*School of Chemistry and Molecular Engineering, East China Normal University, Zhongshan Road North, Shanghai, 200062, P. R. China*

[4]*Department of Microbiology, The University of Hong Kong, Sassoon Road, Hong Kong S.A.R, P. R. China*

[*]*Corresponding should be addressed to H. Sun. E-mail: hsun@hku.hk*

Table of Contents

Supplementary Figures **3**
Supplementary Fig. 1 Separation of Ag^+-binding proteins in *S. aureus* by one dimensional GE-ICP-MS.. 3
Supplementary Fig. 2 Separation of soluble proteins with liquid chromatography.. 4
Supplementary Fig. 3 GE-ICP-MS profiles.. 5
Supplementary Fig. 4 Isothermal titration calorimetry (ITC) results of Ag^+ binding to RpoA, Pgl and 6PGDH. 6
Supplementary Fig. 5 Bioinformatic analysis of identified Ag^+-binding proteins by STRING. 7
Supplementary Fig. 6 Protein-protein interaction network of identified Ag^+-binding proteins.. 8
Supplementary Fig. 7 Measurement of relative gene expression levels with qRT-PCR. 9
Supplementary Fig. 8 Measurement of *in vitro* enzyme activities 10
Supplementary Fig. 9 Measurement of ROS levels with flow cytometry.. 11
Supplementary Fig. 10 Expression levels of Pgl and 6PGDH examined by western blotting.. 12
Supplementary Fig. 11 Comparison of the growth parameters of WT *S. aureus* and gene knockdown strains with and without treatment of Ag^+.. 13
Supplementary Fig. 12 The *2mFo-DFc* polder omit map of Ag^+ coordination sites in Ag-bound 6PGDH. 14
Supplementary Fig. 13 Characterizations of the AgNPs 15
Supplementary Fig. 14 Ag^+/AgNP synergize with antibiotics to kill *S. aureus Newman* 16
Supplementary Fig. 15 The effect of Ag^+ on nascent protein synthesis of *S. aureus*.. 17
Supplementary Fig. 16 Diagram showing that Ag^+ kills *S. aureus* by targeting multiple protein targets and essential pathways.. 18
Supplementary Tables 19
Supplementary Table 1. Summary of identified Ag^+-binding proteins from soluble part of *S. aureus*. 19
Supplementary Table 2. Summary of identified Ag^+-binding proteins from membrane of *S. aureus* 20
Supplementary Table 3. Peptide mass fingerprints of purified 6PGDH. 21
Supplementary Table 4. Peptide mass fingerprints of purified Pgl. 22
Supplementary Table 5. Peptide mass fingerprints of purified RpoA. 23
Supplementary Table 6. Functional Gene Ontology (GO) enrichment of biological process. 24
Supplementary Table 7. Functional GO enrichment of KEGG pathway. 25
Supplementary Table 8. Functional GO enrichment of cellular component. 26
Supplementary Table 9. Summary of X-ray crystallography data collection and refinement statistics. 27
Supplementary Table 10. Ligand-Ag-ligand angle (°) in Ag-6PGDH structure. 28
Supplementary Table 11. Metal-ligand distance (Å) in Ag-6PGDH structure. 29
Supplementary Table 12. Strains, plasmids for protein expression. 30
Supplementary Table 13. Primers for plasmid construction. 31
Supplementary Table 14. Primers for gene knockdown and qRT-PCR. 32
Supplementary Table 15-52. Summary of the peptide mass fingerprints of Ag^+-binding proteins in *S. aureus* 33

Supplementary Figures

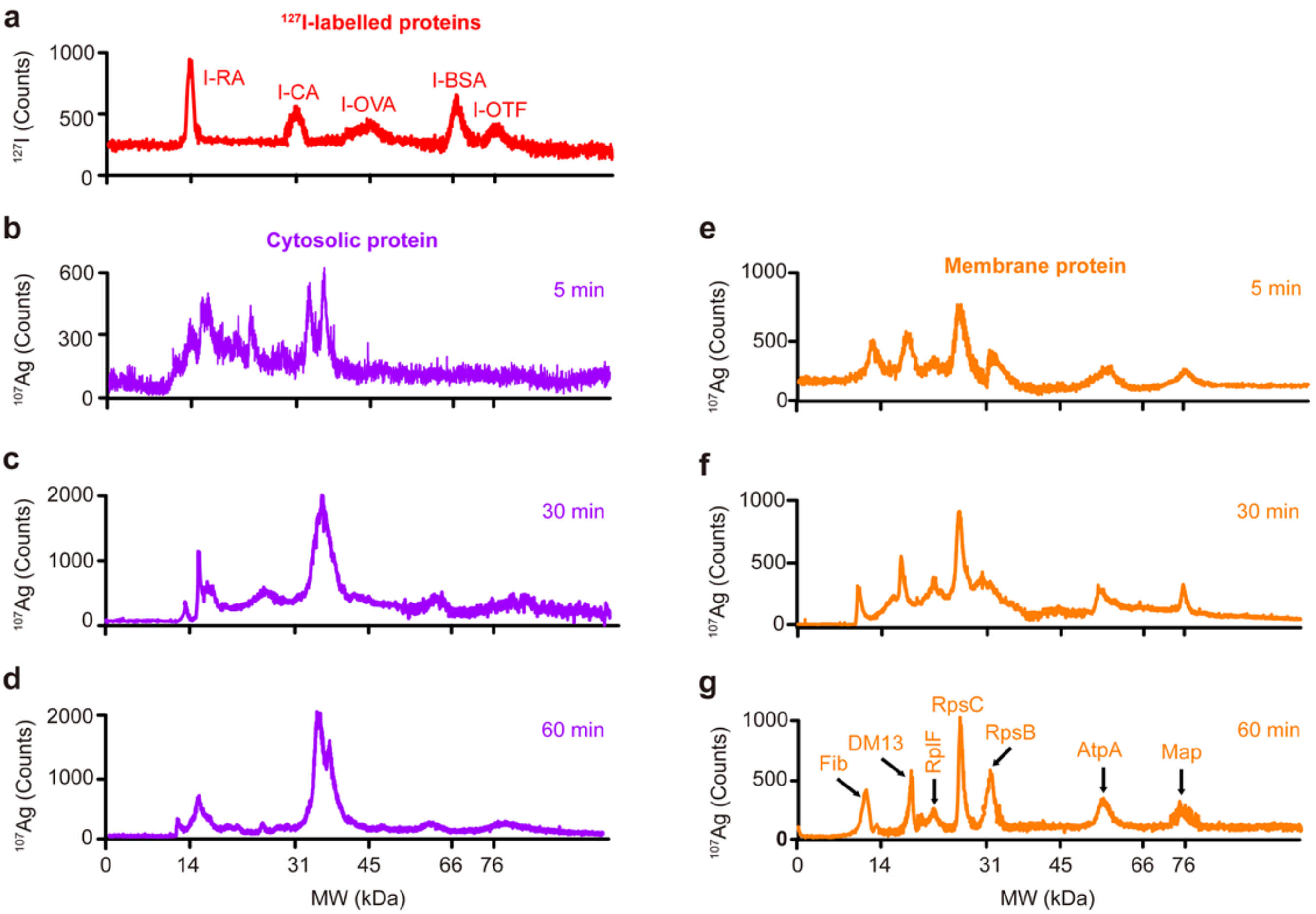

Supplementary Fig. 1 | Separation of Ag^+-binding proteins in *S. aureus* by one dimensional GE-ICP-MS. 1D GE-ICP-MS profiles of (**a**) ^{127}I-labelled standard proteins, Ag^+-binding proteins in (**b-d**) soluble and (**e-g**) membrane fractions of *S. aureus* at different time-points after treatment with 20 µg/mL $AgNO_3$. One representative of three independent replicates is shown (**a-g**).

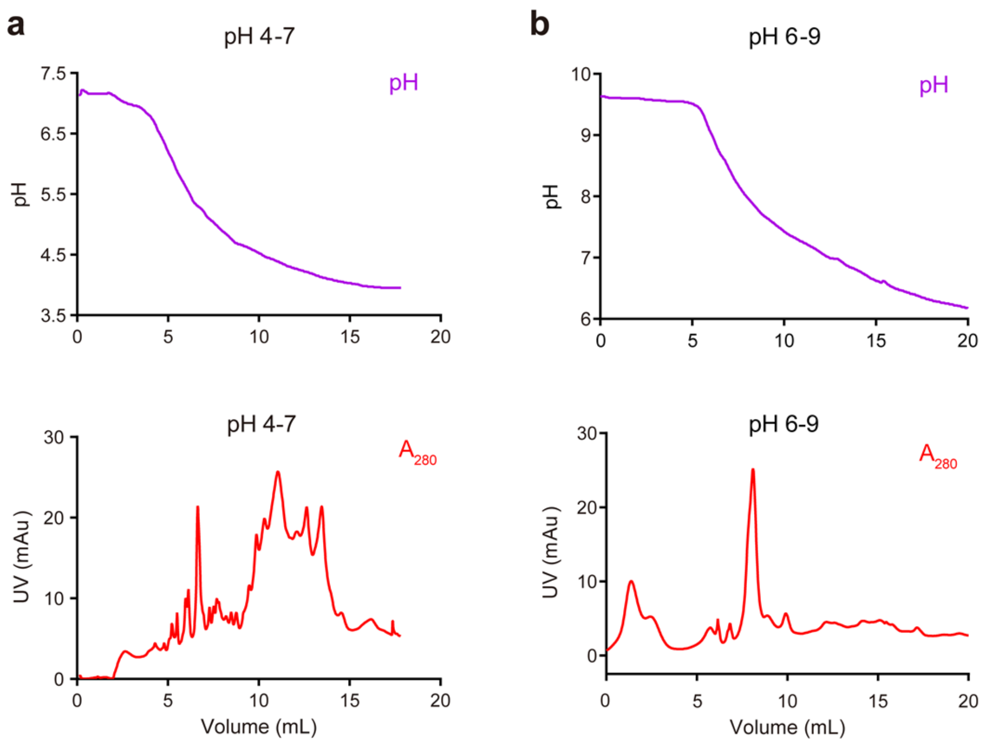

Supplementary Fig. 2 | Separation of soluble proteins with liquid chromatography. Separation of proteins in the pH range of (**a**) 7.0 - 4.0 and (**b**) 9.0 - 6.0 using a Mono P 5/50 GL column. UV absorbance (280 nm) and pH are indicated. The comparison of UV and pH curves manifested that the pI of most proteins in *S. aureus* are in the range of 4 to 6. One representative of three replicates is shown (**a, b**).

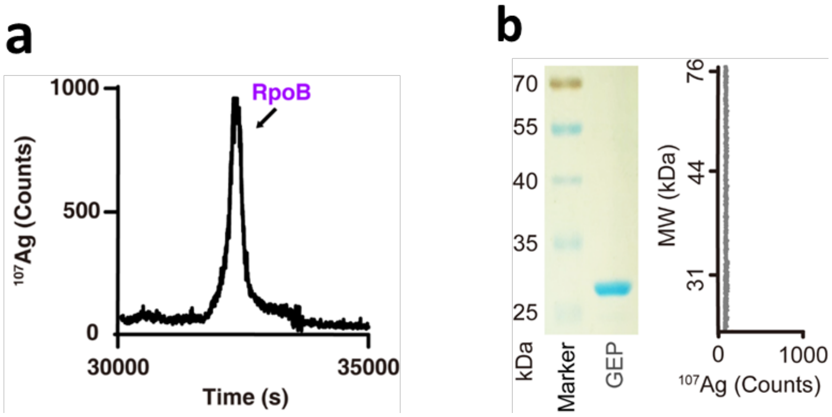

Supplementary Fig. 3 | GE-ICP-MS profiles. (**a**) ^{107}Ag profile corresponding to Ag-bound RpoB at MW of 135.9 kDa. The ^{107}Ag peak corresponding to Ag-RpoB is presented separately as its MW is much higher than other Ag^+-binding proteins. (**b**) GE-ICP-MS profile of purified GEP protein incubated with 5 eq. of Ag^+. One representative of three replicates is shown (**a, b**).

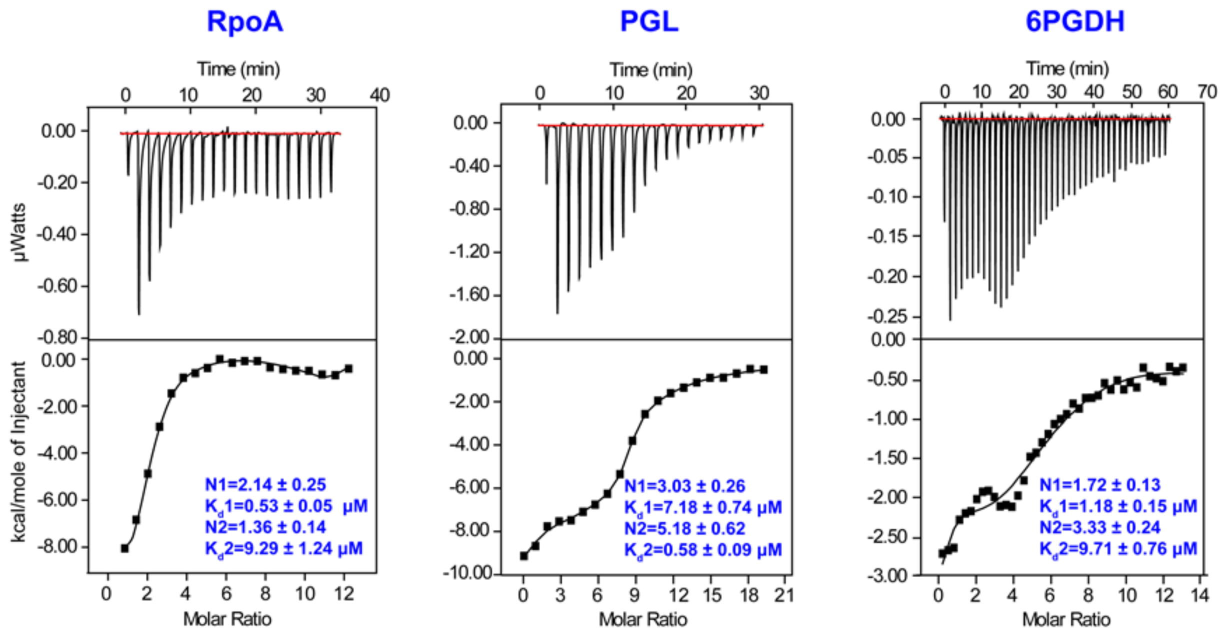

Supplementary Fig. 4 | Isothermal titration calorimetry (ITC) results of Ag^+ binding to RpoA, Pgl and 6PGDH. The titrations were carried out at 25 °C in 35 mM Tris-HNO_3 and 100 mM $NaNO_3$ buffer at pH 7.4. Nitrate rather than other anions is chosen to avoid potential precipitation of Ag^+ ions. The data were fitted into two-set-of-sites binding model using the Origin software. One representative of three replicates is shown.

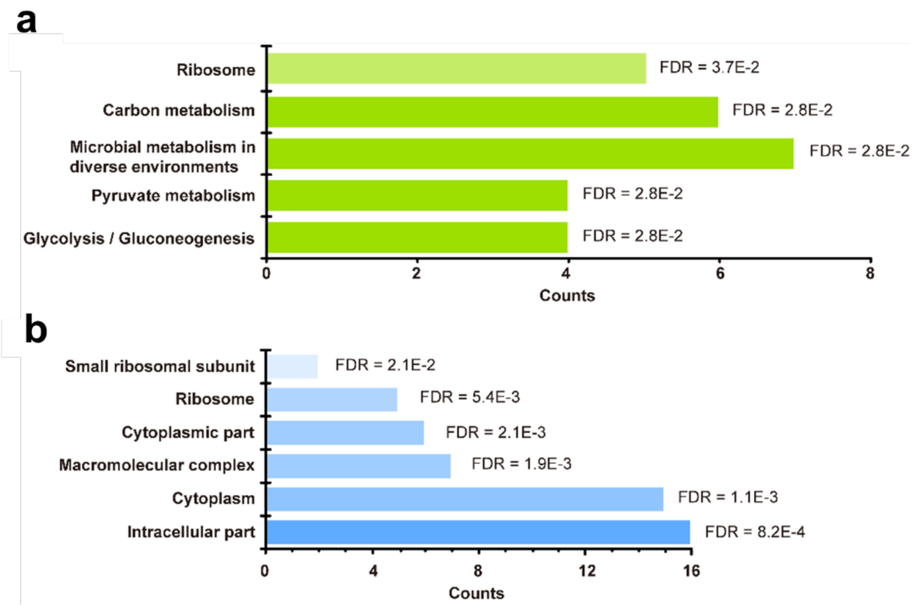

Supplementary Fig. 5 | Bioinformatic analysis of identified Ag^{+}-binding proteins by STRING. (**a**) Functional Gene Ontology (GO) enrichment of KEGG pathways (FDR < 0.05). (**b**) Functional GO enrichment of cellular components. Six cellular components were significantly over presented with FDR < 0.05.

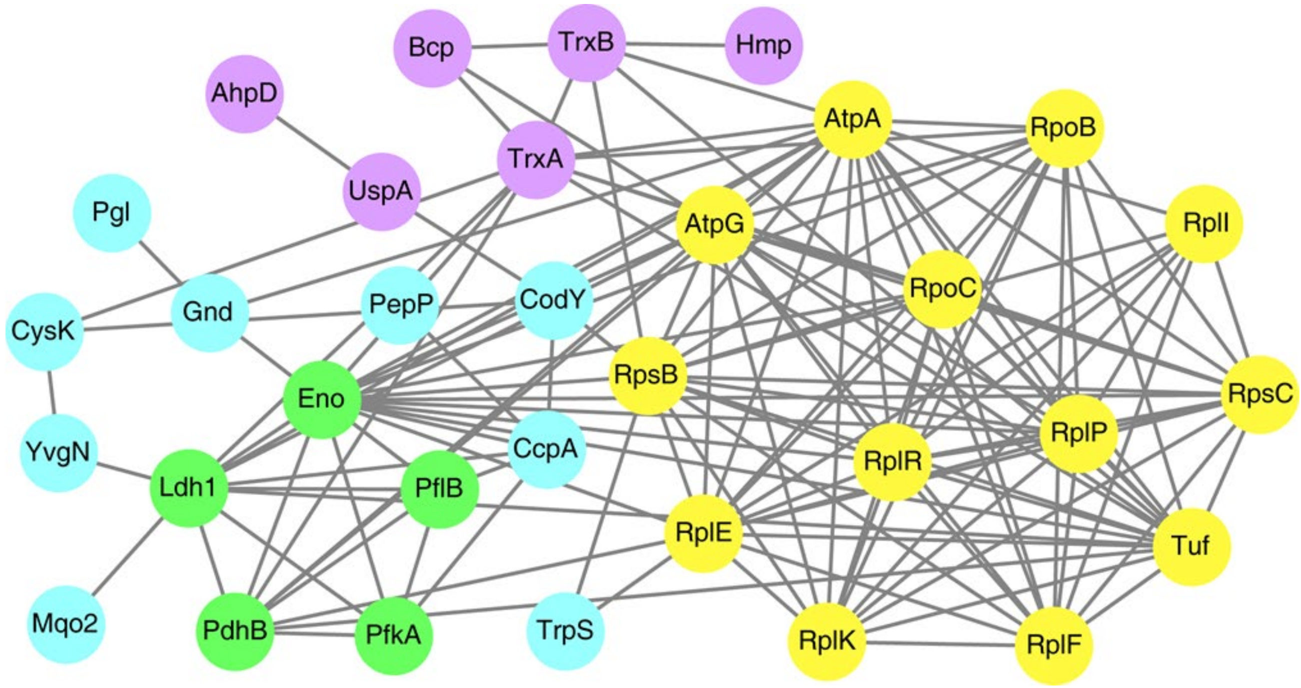

Supplementary Fig. 6 | Protein-protein interaction network of identified Ag^{+}-binding proteins. Protein-protein interactions of identified Ag^{+}-binding proteins revealed by STRING. The Ag^{+}-binding proteins form a highly connected network.

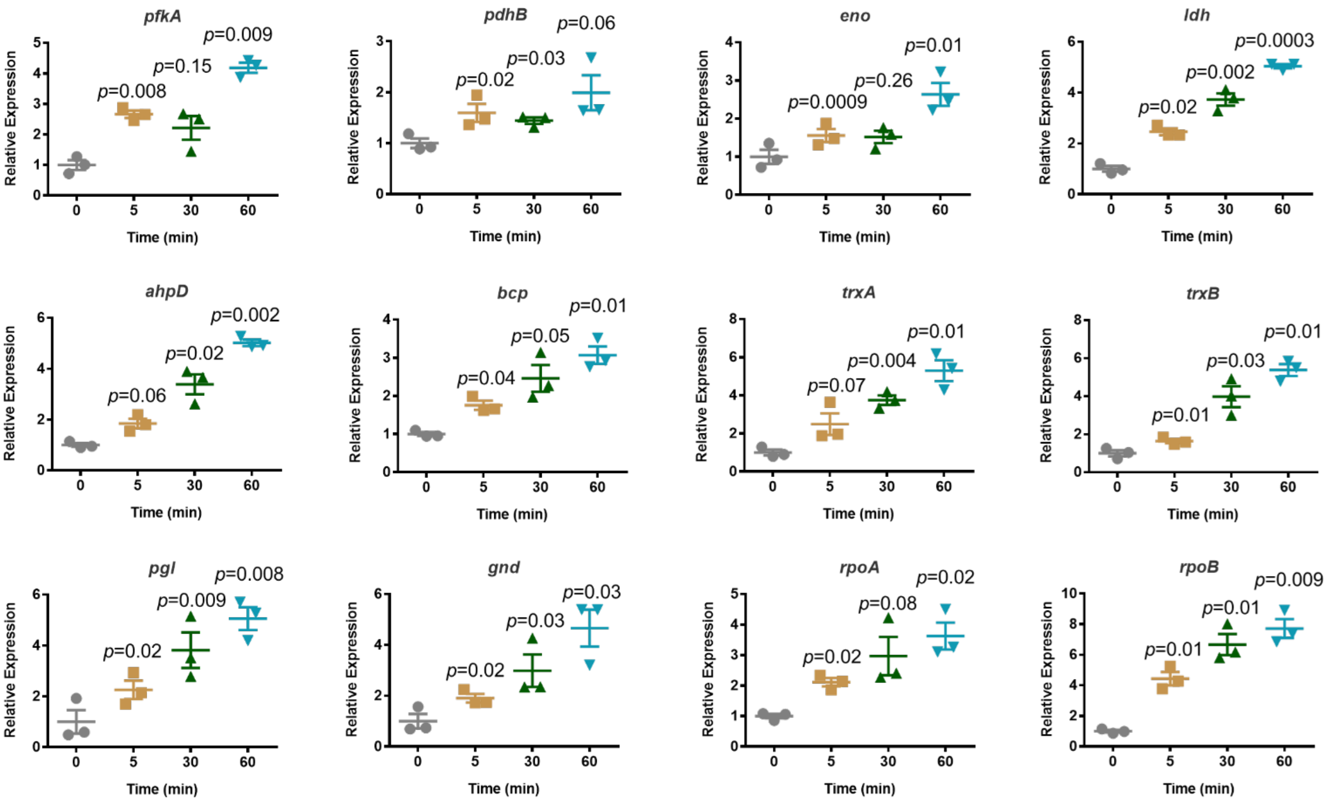

Supplementary Fig. 7 | Measurement of relative gene expression levels with qRT-PCR. Gene expression in S. aureus after treatment of 20 µg/mL of $AgNO_3$ was determined by qPCR and normalized against rrsA and untreated control (n = 3). Mean value of three independent replicates are shown and the error bars indicate ±SEM. Statistical significance was calculated using an unpaired two-tailed Student's t-test compared with control (0 min).

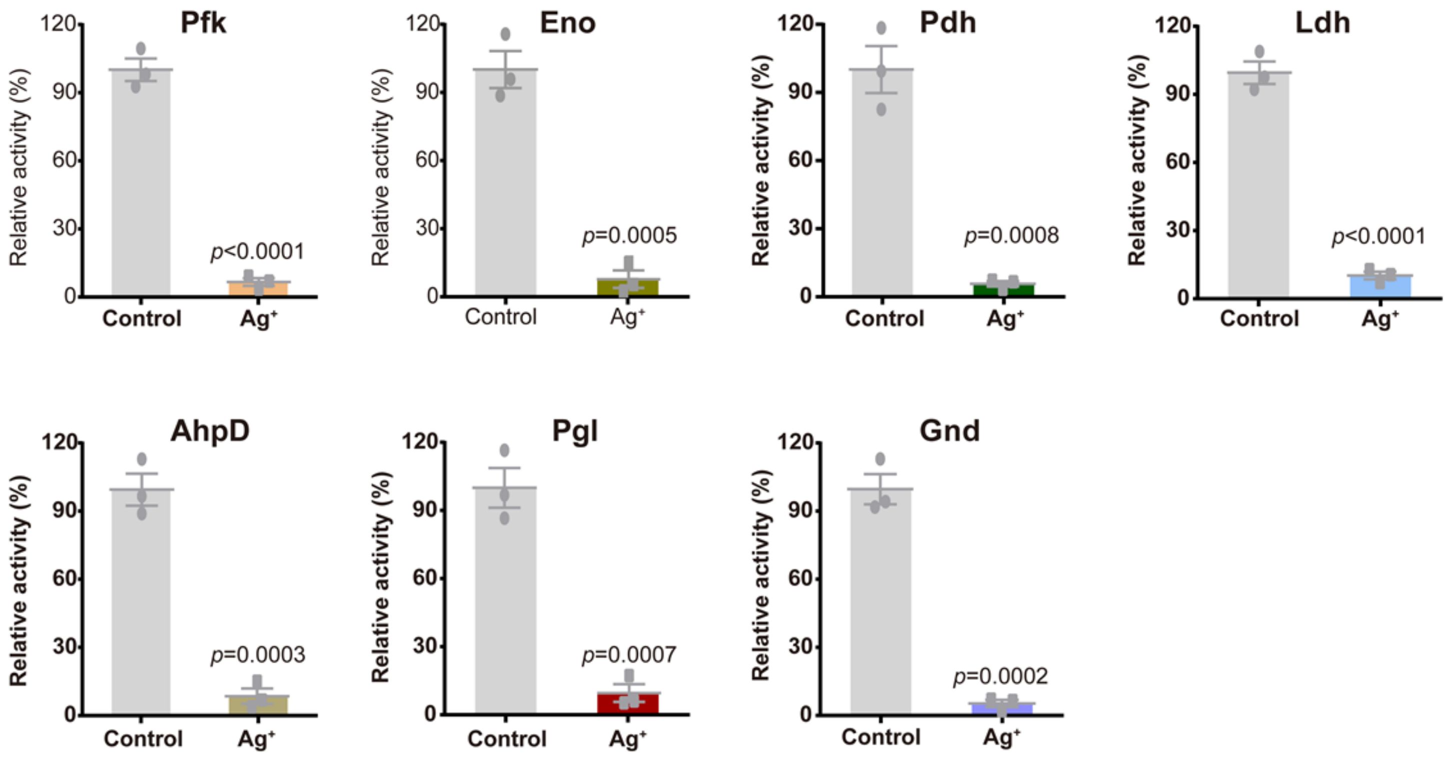

Supplementary Fig. 8 | Measurement of *in vitro* enzyme activities. The enzyme activities were measured in *S. aureus* cell lysate with and without addition of 200 μM $AgNO_3$ (n = 3). Control stands for untreated *S. aureus* cells. Two-tailed t-test was used for all comparisons between two groups. Mean value of three independent replicates are shown and the error bars indicate ±SEM. Statistical significance was calculated using an unpaired two-tailed Student's t-test compared with control.

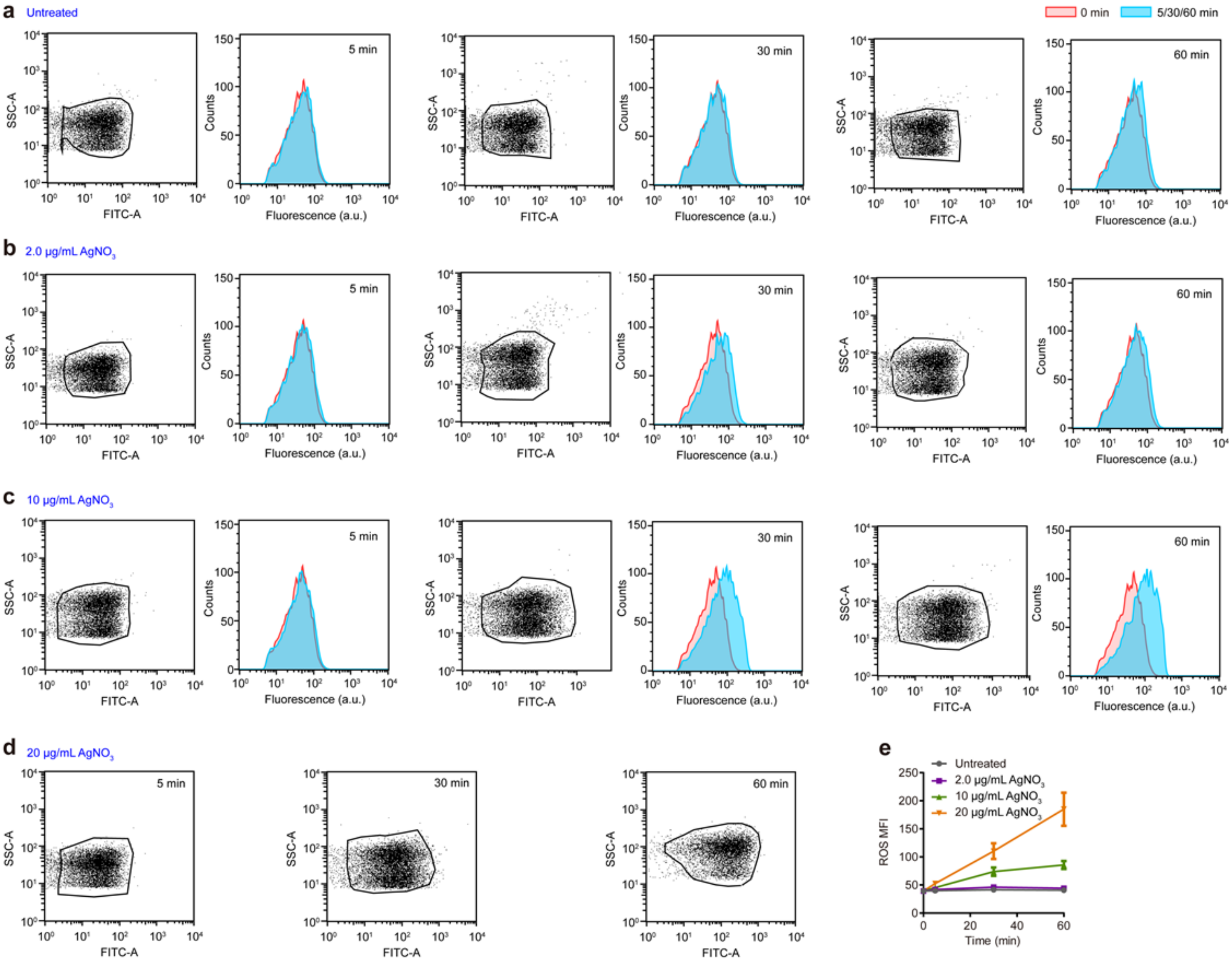

Supplementary Fig. 9 | Measurement of ROS levels with flow cytometry. Gating strategies used for cell sorting and CM-H_2DCFDA fluorescence histogram of *S. aureus* with (red) or without (blue) treatment of Ag^+ (n = 3). (**a**) untreated, (**b**) 2.0 μg/mL $AgNO_3$ treated, and (**c**) 10.0 μg/mL $AgNO_3$ treated. (**d**) 20.0 μg/mL $AgNO_3$ treated. (**e**) Mean fluorescence intensity. Mean value of three replicates are shown and error bars indicate ±SEM (**e**)

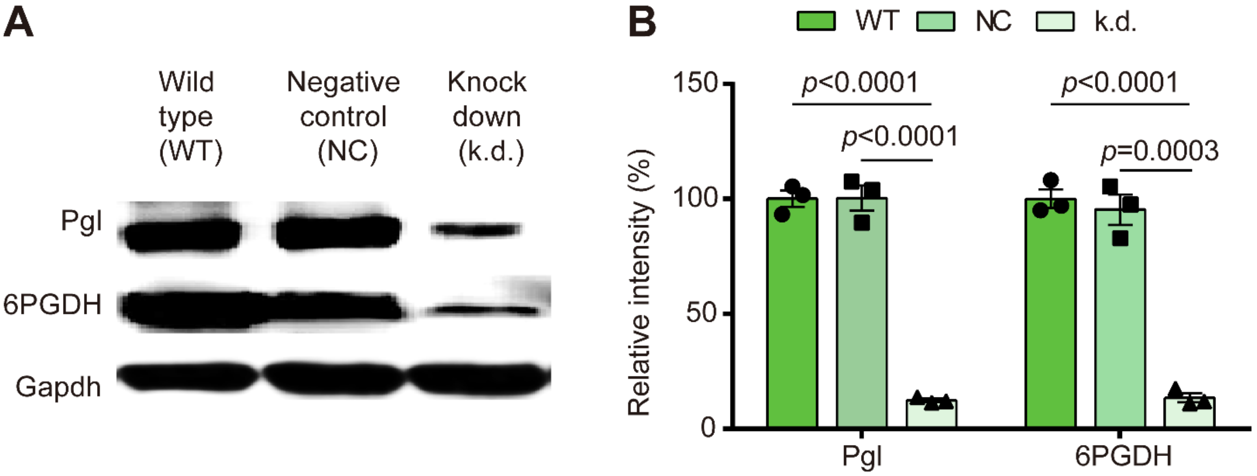

Supplementary Fig. 10 | Expression levels of Pgl and 6PGDH examined by western blotting. The expression levels of Pgl and 6PGDH in wild-type (WT) *S. aureus Newman*, negative control (NC) strains and corresponding knockdown (*k.d.*) strains were examined by western blotting. (**a**) Protein bands obtained by western blotting. One representative of three independent replicates is shown. (**b**) Quantification of the western blot bands (n = 3). Gapdh was used as the reference gene. Two-tailed t-test was used for all comparisons between two groups. Mean value of three independent replicates are shown and error bars indicate ±SEM. Statistical significance was calculated using an unpaired two-tailed Student's t-test compared with WT.

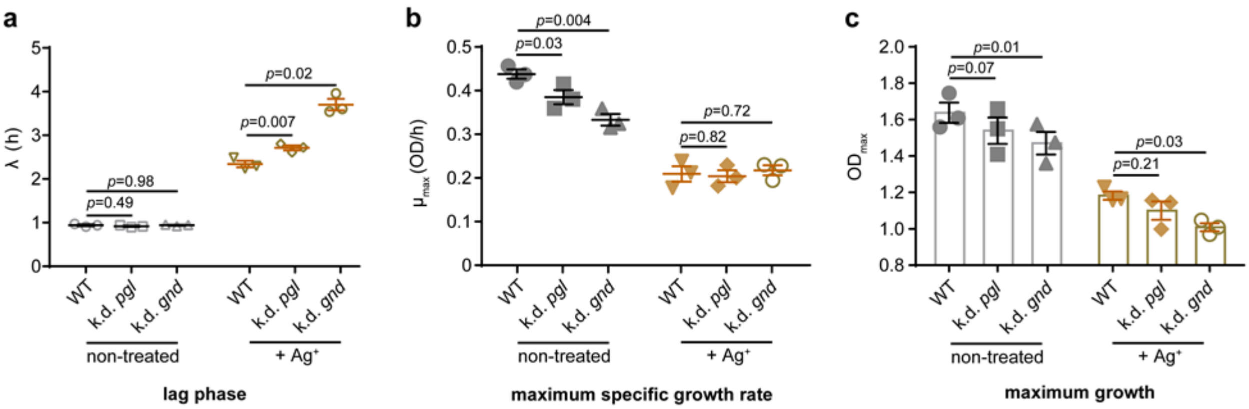

Supplementary Fig. 11 | Comparison of the growth parameters of WT *S. aureus* and gene knockdown strains with and without treatment of Ag^+. (**a**) The lag phase (λ). (**b**) The maximum specific growth rate (μ_{max}). (**c**) The maximum growth (OD_{max}). Mean value of three independent replicates are shown and error bars indicate ±SEM. Statistical significance was calculated using an unpaired two-tailed Student's t-test compared with WT

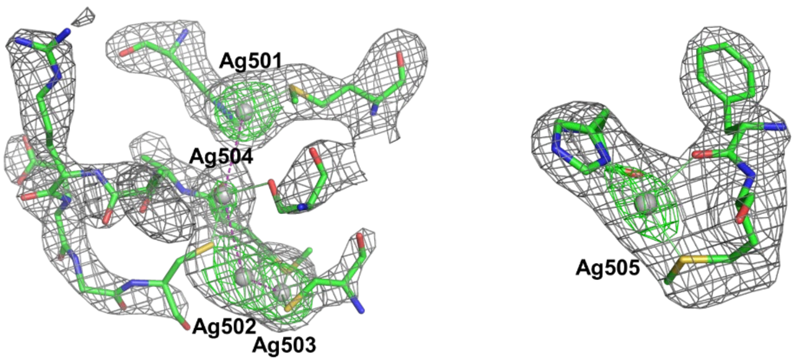

Supplementary Fig. 12 | The *2mFo-DFc* polder omit map of Ag^{+} coordination sites in Ag-bound 6PGDH. The electron densities for Ag (green meshes) are contoured at 7.0σ and those for the surrounding residues (gray meshes) at 3.0σ. The electron density maps were generated by phenix.polder.

a

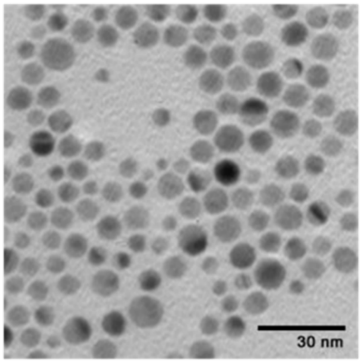

b

Characterization of the AgNPs[a].

Number	Coating materials	Mean diameter	Size distribution (CV)	Hydrodynamic diameter (nm)	Zeta potential (mV)	λ_{max}
N_{10}	PVP	10.1 ± 1.8	< 25%	19	-26	390

[a] Summary of the properties of AgNPs from the website of nanoComposix .

Supplementary Fig. 13 | Characterizations of the AgNPs. (**a**) Images of transmission electron microscopy (TEM) of AgNPs purchased from nanoComposix. One representative of three independent replicates is shown. (**b**) Summary of characterizations of the AgNPs.

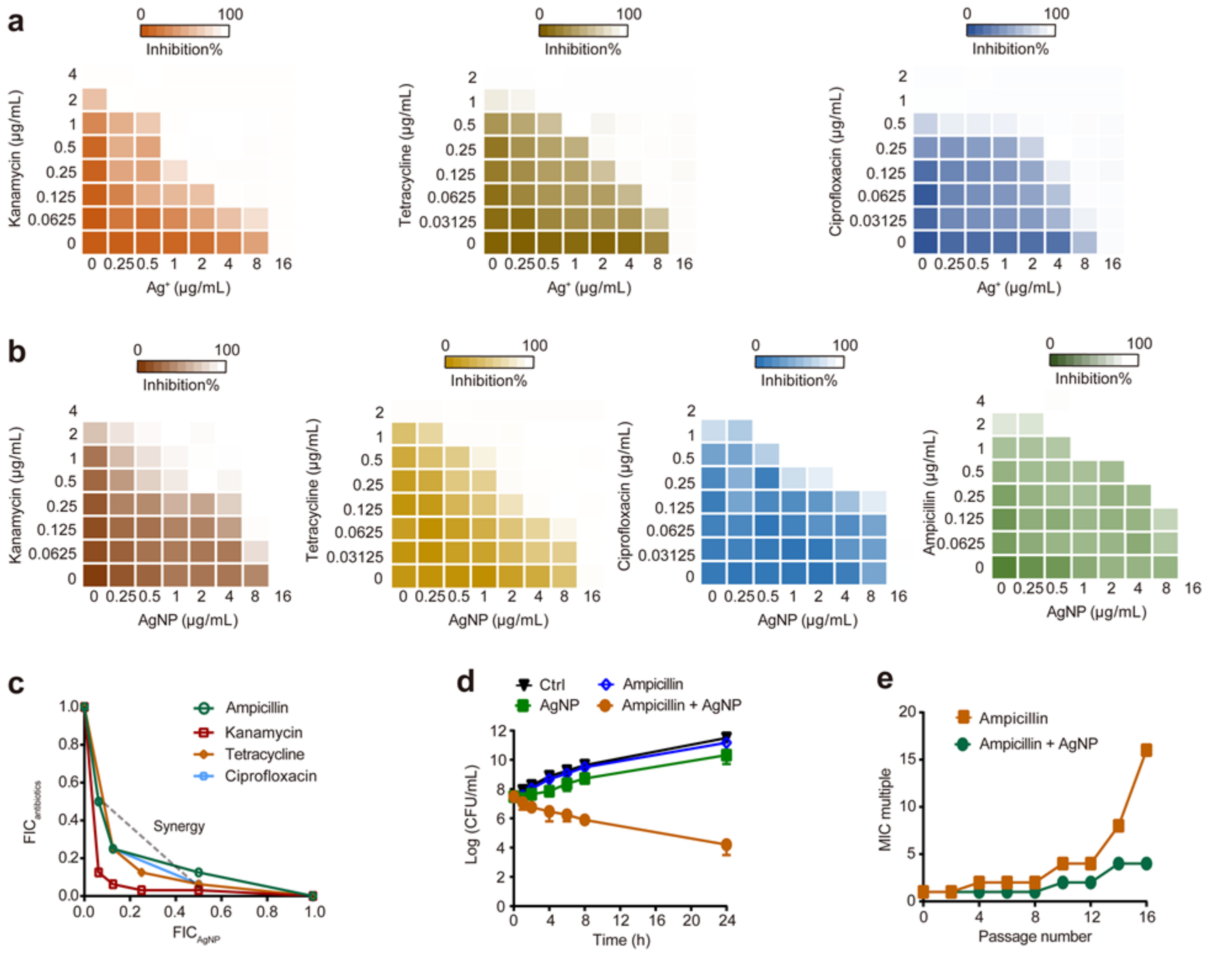

Supplementary Fig. 14 | Ag^{+}/AgNP synergize with antibiotics to kill *S. aureus Newman*. (**a**) Representative heat plot of microdilution checkerboard assay for the combination of antibiotics and Ag^{+} against *S. aureus Newman* (n = 3). (**b**) Representative heat plot of microdilution checkerboard assay for the combination of antibiotics and AgNP against *S. aureus Newman* (n = 3). (**c**) Isobologram of the combination of antibiotic and AgNP against *S. aureus Newman*. (**d**) Time-killing curves for ampicillin and AgNP mono- and combined- therapies against *S. aureus Newman* during 24 hr incubation (n = 3). (**e**) Resistance acquisition curves during serial passage with the subinhibitory concentration of ampicillin or combination of ampicillin and AgNP against *S. aureus Newman* (n = 3). MIC test was performed every two passages. For each experiment, three biological replicates were performed. Mean value of three replicates are shown and error bars indicate ±SEM (**d**).

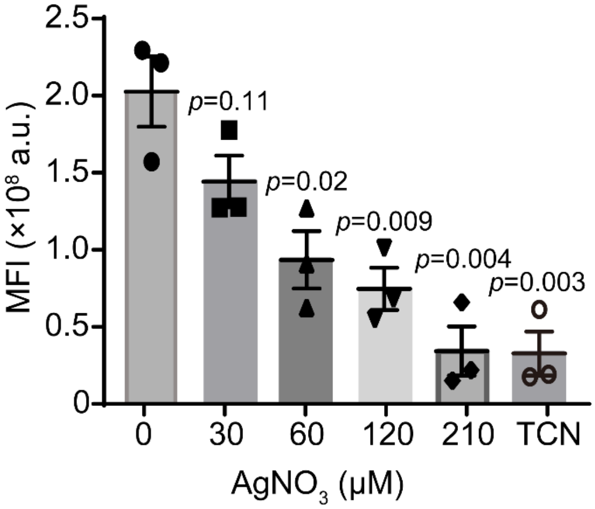

Supplementary Fig. 15 | The effect of Ag^{+} on nascent protein synthesis of S. aureus (n = 3). The antibiotic of tetracycline, which is known to target 30S and disrupt protein synthesis, was used as a control. The fluorescence of S. aureus cells with cell number of 1 OD was recorded. Mean value of three independent replicates are shown and the error bars indicate ±SEM. Statistical significance was calculated using an unpaired two-tailed Student's t-test compared with untreated group.

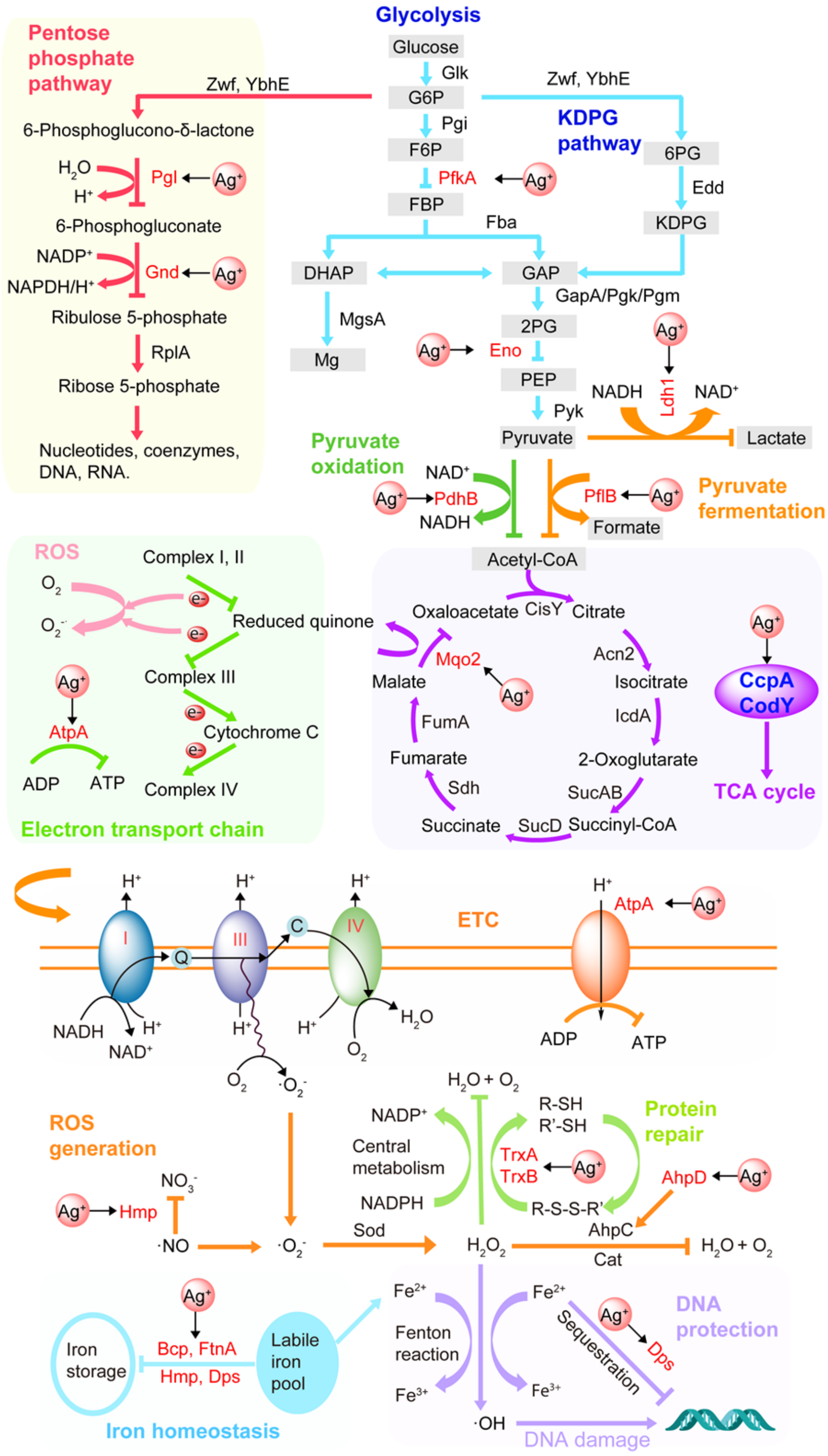

Supplementary Fig. 16 | Diagram showing that Ag^+ kills *S. aureus* by targeting multiple protein targets and essential pathways.

Supplementary Tables

Supplementary Table 1. Summary of identified Ag^{+}-binding proteins from soluble part of *S. aureus*.

Gene	Gene product	Accession No.	pI	MW/ kDa	Protein Score	Protein Score C.I.%	Peptide count
UPF0356	UPF0356 protein	gi|487385298	5.17	8.7	104	99.9	5
AhpD	Alkylhydroperoxidase AhpD family core domain protein	gi|377718914	5.34	14.8	67	94.9	4
RplK	50S ribosomal protein L11	gi|613363411	8.71	14.9	67	94.7	4
RplI	50S ribosomal protein L9	gi|583108401	9.28	16.3	129	100	12
AhpD	Alkylhydroperoxidase AhpD	gi|447069508	5.57	16.6	220	100	8
Bcp	Bacterioferritin comigratory protein	gi|49486681	5.54	17.2	72	98.6	13
Dps	General stress protein (Dps family protein)	gi|584762748	4.98	18.0	328	100	6
UspA	Universal stress protein UspA	gi|446556828	5.45	19.1	381	100	8
FtnA	Ferritin	gi|582593655	4.68	19.5	94	99.9	6
Tnp	Transposase	gi|537468406	6.43	20.2	163	100	7
TrxA	Thioredoxin	gi|446104989	5.28	21.9	100	99.9	7
CodY	Transcriptional repressor CodY	gi|686242013	5.57	28.7	188	100	8
RpoA	DNA-directed RNA polymerase subunit alpha (partial)	gi|15927804	4.98	32.4	246	100	12
CysK	Cysteine synthase	gi|445979740	5.45	33.0	127	100	5
TrxB	Thioredoxin reductase	gi|446057106	5.21	33.7	204	100	11
Ldh1	Lactate dehydrogenase	gi|446954621	4.95	34.6	111	100	9
PfkA	6-Phosphofructokinase	gi|446640221	5.92	35.1	124	100	9
PdhB	2-Oxoisovalerate dehydrogenase (Pyruvate dehydrogenase E1 component subunit beta)	gi|872535879	4.65	35.3	214	100	8
CcpA	Catabolite control protein A	gi|686335442	5.46	36.1	163	100	8
TrpS	Tryptophanyl-tRNA synthetase	gi|570299737	6.47	36.7	148	100	9
Pgl	6-Phosphogluconolactonase	gi|379021683	5.04	38.9	139	100	12
PepP	Peptidase M24	gi|686441981	5.23	39.5	120	100	8
Int	Integrase	gi|581947497	9.73	40.1	73	98.7	12
Hmp	Flavohemoprotein	gi|581611265	5.17	43.0	139	100	11
TelA	Tellurite resistance protein TelA	gi|657833828	5.64	43.3	203	100	12
Tuf	Elongation factor Tu	gi|446963312	4.74	43.1	302	100	14
Eno	Alpha-enolase	gi|555636526	4.58	47.2	276	100	15
Gnd	6-Phosphogluconate dehydrogenase, decarboxylating	gi|487390600	5.00	51.9	149	100	14
Mqo2	Malate:quinone oxidoreductase	gi|447052793	6.02	56.1	202	100	14
PflB	Formate acetyltransferase	gi|446817403	5.31	85.3	111	100	16
RpoB	DNA-directed RNA polymerase subunit beta	gi|578916640	6.40	135.9	326	100	12

Supplementary Table 2. Summary of identified Ag^{+}-binding proteins from membrane of *S. aureus*.

Gene	Protein Name	Accession No.	Theoretical MW/kDa	Protein Score	Protein Score C. I.%	Peptide count
Fib	Fibrinogen-binding protein	gi|757461200	12.9	206	100	7
DM13	Electron transfer DM13	gi|447016303	16.2	113	100	7
RplF	50S ribosomal protein L6	gi|686128415	19.8	197	100	10
RpsC	30S ribosomal protein S3	gi|686391303	24.1	224	100	10
RpsB	30S ribosomal protein S2	gi|686127623	29.1	170	100	9
AtpA	ATP synthase subunit alpha	gi|446897627	54.6	190	100	11
Map	MHC class II analog protein	gi|375369277	77.0	245	100	22

Supplementary Table 3. Peptide mass fingerprints of purified 6PGDH.

Protein Name		Accession No.	Protein Score	Protein Score C.I. %	Protein MW/Da	Peptide Count
Phosphogluconate dehydrogenase		WP_020807976.1	361	100	51955.2	15
**Calc. Mass**	**Obs. Mass**	**Peptide Seq.**		**Modification**	**Ion Score**	**C.I. %**
1028.516	1028.577	GYSVSVFNR				
1028.516	1028.577	GYSVSVFNR			35	
1074.5765	1074.6427	LGDLAMIWR				
1090.5714	1090.6367	LGDLAMIWR		Oxidation (M)[6]		
1090.5714	1090.6367	LGDLAMIWR		Oxidation (M)[6]	22	
1102.564	1102.6272	NLAWNIESR				
1102.564	1102.6272	NLAWNIESR			50	
1108.5997	1108.6572	FISSIKEER				
1258.5488	1258.616	DYFGAHTYER				
1258.5488	1258.616	DYFGAHTYER			53	
1267.5988	1267.7146	DGASCVTYIGPK		Carbamidomethyl (C)[5]		
1321.6747	1321.7419	NIVTEYQDALR				
1333.6383	1333.7468	KASEDNEWNLK				
1380.7594	1380.83	AADLPANLIQAQR				
1380.7594	1380.83	AADLPANLIQAQR			101	
1386.6437	1386.7128	DYFGAHTYERK				
1431.6508	1431.7213	ICSYAQGFAQMR		Carbamidomethyl (C)[2]		
1447.6458	1447.7229	ICSYAQGFAQMR		Carbamidomethyl (C)[2], Oxidation (M)[11]		
2273.156	2273.2661	NIHPTYSLEEFVNSLEKPR				
2401.251	2401.3545	NIHPTYSLEEFVNSLEKPRK				
2458.2725	2458.3616	GKNIHPTYSLEEFVNSLEKPR				
2893.364	2893.4949	DVVATGVQNGVPTPGFS SSINYYDSYR				

Supplementary Table 4. Peptide mass fingerprints of purified Pgl.

**Protein Name**		**Accession No.**	**Protein Score**	**Protein Score C.I. %**	**Protein MW/Da**	**Peptide Count**
Hypothetical protein PGL		WP_061652824.1	427	100	38879.163	15
**Calc. Mass**	**Obs. Mass**	**Peptide Seq.**		**Modification**	**Ion Score**	**C.I. %**
1136.5331	1136.5999	FELNENQSR				
1179.6005	1179.669	YVAVTDLGADR				
1471.7944	1471.8934	YLFEAVYGAGIIR				
1542.7217	1542.808	AGTGCYVSISEDKR		Carbamidomethyl (C)[5]		
1576.7788	1576.871	MYELNTHTGEIIR				
1576.7788	1576.871	MYELNTHTGEIIR			2	
1592.7737	1592.8627	MYELNTHTGEIIR		Oxidation (M)[1]		
1606.7972	1606.8923	LSHDQQFLYVSNR				
1606.7972	1606.8923	LSHDQQFLYVSNR			73	
1625.8031	1625.8923	GIYRFELNENQSR				
1625.8031	1625.8923	GIYRFELNENQSR				
1783.9589	1784.041	YVAVTDLGADRIVTYK				
1863.9348	1864.0454	LIQELAHDFPTGTHER				
1863.9348	1864.0454	LIQELAHDFPTGTHER			54	
1997.0452	1997.1409	HLTIPENFDGDTKLAAVR				
2069.0801	2069.1978	IDLLETGFELEASTYLVR				
2410.2361	2410.3894	VLDNGQHLELVTITESGG QFPR				
2410.2361	2410.3894	VLDNGQHLELVTITESGG QFPR			147	
2575.3052	2575.4392	LSHDQQFLYVSNRGHDSI AIFK				
3037.4209	3037.6101	DFNIASSDDLLVCAHEQG DSVLTVFER		Carbamidomethyl (C)[13]		
3145.5112	3145.7017	FAYVVHELSNTVSVAEYN DGKFEELER				

Supplementary Table 5. Peptide mass fingerprints of purified RpoA.

Protein Name		Accession No.	Protein Score	Protein Score C.I. %	Protein MW/Da	Peptide Count
DNA-directed RNA polymerase subunit alpha		WP_002461762.1	359	100	35033.160	15
**Calc. Mass**	**Obs. Mass**	**Peptide Seq.**		**Modification**	**Ion Score**	**C.I. %**
940.4669	940.5332	SYNCLKR		Carbamidomethyl (C)[4]		
940.4669	940.5332	SYNCLKR		Carbamidomethyl (C)[4]	26	
985.5676	985.6354	LEDLGLGLR				
988.5462	988.6208	FVVEPLER			66	
1031.5554	1031.6343	MIEIEKPR		Oxidation (M)[1]		
1095.543	1095.6351	VNYTVENTR				
1095.543	1095.6351	VNYTVENTR				
1138.5852	1138.6724	GYGTTLGNSLR				
1138.5852	1138.6724	GYGTTLGNSLR			81	
1168.73	1168.8127	ILLSSLPGAAVK				
1247.6366	1247.7397	IETIEISEDAK				
1276.726	1276.822	YKLEDLGLGLR				
1294.6863	1294.7892	GYGTTLGNSLRR				
1320.7311	1320.8237	FGKFVVEPLER				
1324.8312	1324.9213	RILLSSLPGAAVK				
1324.8312	1324.9213	RILLSSLPGAAVK				
1648.8462	1648.9722	VLEMSIEELDLSVR		Oxidation (M)[4]		
1709.8593	1709.9854	IYSEEDKTLEIDVR				
2260.1741	2260.3438	MIEIEKPRIETIEISEDAK		Oxidation (M)[1]		

Supplementary Table 6. Functional Gene Ontology (GO) enrichment of biological process.

GO ID	GO term	Count	FDR*	Protein associated
GO:0006096	Glycolytic process	4	4.0E-3	Eno; Ldh1; PdhB; PfkA
GO:0046034	ATP metabolic process	5	4.0E-3	AtpA; Eno; Ldh1; PdhB; PfkA
GO:0044723	Single-organism carbohydrate metabolic process	6	5.3E-3	Eno; Ldh1; PdhB; PfkA; PflB; 6PGDH
GO:0034641	Cellular nitrogen compound metabolic process	17	6.7E-3	AtpA; CodY; Eno; Ldh1; PdhB; PfkA; RplF; RplI; RplK; RpoB; RpsB; RpsC; Tuf; CcpA; CysK; 6PGDH; TrpS
GO:0055114	Oxidation-reduction process (response to oxidative stress)	5	8.5E-3	Bcp; AhpD; Dps; TrxA; TrxB
GO:0006412	Translation	7	8.6E-3	RplF; RplI; RplK; RpsB; RpsC; Tuf; TrpS
GO:1901564	Organonitrogen compound metabolic process	11	1.0E-2	AtpA; Eno; Ldh1; PdhB; PfkA; RplF; RplI; RplK; RpsB; RpsC; Tuf; CcpA; 6PGDH
GO:0044238	Primary metabolic process	14	2.6E-2	AtpA; CodY; Eno; Ldh1; PdhB; PfkA; PflB; RplF; RplI; RplK; RpoB; RpsB; RpsC; Tuf
GO:0010467	Gene expression	10	2.8E-2	RpoA; RplF; RplI; RplK; RpoB; RpsB; RpsC; Tuf; CcpA; TrpS
GO:0044271	Cellular nitrogen compound biosynthetic process	11	3.0E-2	AtpA; CodY; RplF; RplI; RplK; RpoB; RpsB; RpsC; Tuf; CcpA; TrpS
GO:0034645	Cellular macromolecule biosynthetic process	10	3.0E-2	CodY; RplF; RplI; RplK; RpoB; RpsB; RpsC; Tuf; CcpA; TrpS

* FDR denotes false discovery rate.

Supplementary Table 7. Functional GO enrichment of KEGG pathway.

Pathway ID	GO term	Count	FDR	Protein associated
10	Glycolysis/Gluconeogenesis	4	2.8E-2	Eno; Ldh1; PdhB; PfkA
620	Pyruvate metabolism	4	2.8E-2	Mqo2; Ldh1; PdhB; PflB
1120	Microbial metabolism in diverse environments	7	2.8E-2	CysK; 6PGDH; Mqo2; Eno; Ldh1; PdhB; PfkA
1200	Carbon metabolism	6	2.8E-2	CysK; 6PGDH; Mqo2; Eno; PdhB; PfkA
3010	Ribosome	5	3.7E-2	RplF; RplI; RplK; RpsB; RpsC

Supplementary Table 8. Functional GO enrichment of cellular component.

GO ID	GO term	Count	FDR	Protein associated
GO:0005622	Intracellular part	16	8.2E-4	UspA; AtpA; CodY; Eno; FtnA; Ldh1; PfkA; PflB; RplF; RplI; RplK; RpsB; RpsC; Tuf; TrpS, TrxB
GO:0005737	Cytoplasm	15	1.1E-3	UspA; CodY; Eno; FtnA; Ldh1; PfkA; PflB; RplF; RplI; RplK; RpsB; RpsC; Tuf; TrpS, TrxB
GO:0032991	Macromolecular complex	7	1.9E-3	AtpA; Eno; RplF; RplI; RplK; RpsB; RpsC
GO:0044444	Cytoplasmic part	6	2.1E-3	Eno; RplF; RplI; RplK; RpsB; RpsC
GO:0005840	Ribosome	5	5.4E-3	RplF; RplI; RplK; RpsB; RpsC
GO:0015935	Small ribosomal subunit	2	2.1E-2	RpsB; RpsC

Supplementary Table 9. Summary of X-ray crystallography data collection and refinement statistics.

**Data collection**	**Ag-6PGDH**	**6PG-6PGDH**
**PDB ID**	**7CB6**	**7CB5**
Wavelength (Å)	0.97918	0.97918
Resolution range (Å)	80.27 - 2.64 (2.71-2.64)	80.84 - 2.54 (2.61-2.54)
Space group	$P2_12_12_1$	$P2_12_12_1$
a, b, c (Å)	90.86, 133.86, 171.32	91.92, 132.62, 169.85
α, β, γ (º)	90.00, 90.00, 90.00	90.00, 90.00, 90.00
Unique reflections	61985 (4549)	69042 (5067)
Rmerge(I)	0.132 (1.029)	0.113 (1.338)
Rpim(I)	0.036 (0.351)	0.032 (0.368)
CC1/2	0.999 (0.796)	0.999 (0.814)
Completeness (%)	99.78 (99.99)	99.81 (99.96)
Mean I/sigma(I)	15.3 (2.7)	17.2 (3.4)
Redundancy	13.4 (14.1)	13.5 (13.9)
Wilson B-factor	56.0	54.2
Average B-factor	64.60	71.37
Average B-factor (protein)	64.59	71.49
Average B-factor (all ligands)	81.18	72.96
Average B-factor (Ag)	70.37	-
Reflections for refinement	61985 (4549)	69042 (5067)
R-work	0.205 (0.271)	0.199 (0.303)
R-free	0.246 (0.301)	0.241 (0.304)
Number of non-hydrogen atoms	14379	14529
RMSD (bonds) (Å)	0.002	0.003
RMSD (angles) (º)	0.449	0.547
Ramachandran favoured (%)	95.63	95.53
Ramachandran allowed (%)	4.15	4.37
Ramachandran outliers (%)	0.22	0.11

Supplementary Table 10. Ligand-Ag-ligand angle (°) in Ag-6PGDH structure.

Atom1	Atom2	Atom3	Angle (°)
Met140/Sδ	Ag501	His185/Nδ1	131.5
Cys168/Sγ	Ag502	Cys363/Sγ	157.8
Cys168/Sγ	Ag503	Met357/Sδ	166.0
Cys363/Sγ	Ag504	Ser138/Oγ	118.1
Cys363/Sγ	Ag504	Met357/O	90.6
Ser138/Oγ	Ag504	Met357/O	127.9
His211/Nδ1	Ag505	Met239/Sδ	168.8

*The measurements are based on the atomic coordinates for the polymeric Chain A and Ag atoms or water molecules.

Supplementary Table 11. Metal-ligand distance (Å) in Ag-6PGDH structure.

Atom1	Atom2	Distance (Å)
Ag501	Met140/Sδ	2.6
Ag501	His185/Nδ1	2.2
Ag502	Cys363/Sγ	2.4
Ag502	Cys168/Sγ	2.4
Ag502	Ag473	2.8
Ag503	Cys168/Sγ	2.5
Ag503	Met357/Sδ	2.6
Ag504	Met357/O	2.3
Ag504	Ser138/Oγ	3.0
Ag504	Trp359/N	3.2
Ag505	His211/Nδ1	2.2
Ag505	Met239/Sδ	2.5

Supplementary Table 12. Strains, plasmids for protein expression.

**Strains**	**Application**
XL1-Blue	Plasmid maintenance
BL21(DE3)	Protein expression
**Plasmids**	
pHisSUMO	
pHisSUMO-*6PGDH*	Wild-type 6PGDH protein expression
pHisSUMO-*6PGDH*^{C168S}	6PGDH^{C168S} mutant protein expression
pHisSUMO-*6PGDH*^{C330S}	6PGDH^{C330S} mutant protein expression
pHisSUMO-*6PGDH*^{C363S}	6PGDH^{C363S} mutant protein expression
pHisSUMO-*6PGDH*^{M357S}	6PGDH^{M357S} mutant protein expression
pHisSUMO-*6PGDH*^{M140S}	6PGDH^{M140S} mutant protein expression
pHisSUMO-*6PGDH*^{M239S}	6PGDH^{M239S} mutant protein expression
pHisSUMO-*6PGDH*^{H185S}	6PGDH^{H185S} mutant protein expression
pHisSUMO-*6PGDH*^{H211S}	6PGDH^{H211S} mutant protein expression
pHisSUMO-*pgl*	Wild-type Pgl protein expression
pHisSUMO-*rpoA*	Wild-type RpoA protein expression

Supplementary Table 13. Primers for plasmid construction.

	**Forward Primers (AgeI)**	**Reverse Primers (EcoRI)**
6PGDH	GAATTCACCGGTGGAATGACACAACAAATTG	CCGGAATTCTTATTCTTCAATCC
Pgl	GAATTCACCGGTGGAATGATGACAAATGGATATATTG	CCGGAATTCTTAAAATATGACAC
RpoA	GAATTCACCGGTGGAATGATAGAAATCGAA	CCGGAATTCTCAATCTTCTTTTC
6PGDH ^{C168S}	CAAAAGACGGTGCTTCAAGCGTTACTTATA	GGTCCAATATAAGTAACGCTTGAAGCACCG
6PGDH^{C330S}	TATATGAGTAAAATTAGCTCTTATGCAC	ACCTTGTGCATAAGAGCTAATTTTACTC
6PGDH^{C363S}	GATTTGGAGAGAAGGTAGCATTATCCGTG	TTGTGCACGGATAATGCTACCTTCTCTC
6PGDH^{M357S}	CTTGGTGATTTAGCTAGCATTTGGAG	CCTTCTCTCCAAATGCTAGCTAAATC
6PGDH^{M140S}	CTGGTCCGTCATTAAGCCCGGGTGGAC	CTTCTTGTCCACCCGGGCTTAATGACGG
6PGDH^{M239S}	CACTGGTGATATTTTCAGCAAATGGAATGC	CACCAGCATTCCATTTGCTGAAAATATC
6PGDH^{H185S}	CTATGTGAAAATGGTAAGCAATGGTATCG	GCATATTCGATACCATTGCTTACCATTTTC
6PGDH^{H211S}	ATTATTAGGCATGTCTAGCGAAGACATTG	GTTTGAGCAATGTCTTCGCTAGACATGCC

Supplementary Table 14. Primers for gene knockdown and qRT-PCR

**Knock down**	**Forward Primers**	**Reverse Primers**
gnd	CTAGTAAGTAATATGACACAACAAAT	AACATTTGTTGTGTCATATTACTTAC
pgl	CTAGTTACTGCAACGTACTTACCATC	AACGATGGTAAGTACGTTGCAGTAAC
**qRT-PCR**		
ahpD	GTCTGTAATGAGCCAAGGTGAA	GGATATCAACACAATAATGACAACCG
bcp	TACCACAGAAGCTTGTGACTTTA	CGAAATTCAATCCGTGTTTCTCA
trxA	TGATCGCTCCGGTATTAGAAG	TTTAGCTGCAGTTGATGGATTT
trxB	TGCTACAGGTGCAGAATACAAG	CACCACCACCGATAACGAATAG
rpoA	GTACGTGATGAAGGCGAAGT	GCGTAACCTCTACCCTTGTTAG
rpoB	GTGATTCTGGTGCGGCTATTA	CGCCGTTCTCTTCAACTAGAC
ldh	TCGCAACGTCGTAGAAATCC	AAGTTCTTCTGCTTCAGCCATA
pfkA	GTCCAGAGTTTAAGGAGCAAGA	ACTACCGTCACCACCAATAAC
pdhB	TGGTTTAGCGATGGGTCTTG	GTCCAGCAATCGCATCAAATAC
eno	CCGTTCAGGTGAAACAGAAGA	GCAATACGGTCAGTACGTGATAA
pgl	AGCTGGTACAGGTTGTTATGT	AATTTCACCTGTGTGCGTATTT
gnd	TGTACCAACACCTGGTTTCTC	CGTATGAGCACCGAAGTAGTC

Supplementary Table 15-52. Summary of the peptide mass fingerprints of Ag^{+}-binding proteins in *S. aureus*

Supplementary Table 15. Peptide mass fingerprints of Int

**Protein Name**		**Accession No.**	**Protein Score**	**Protein Score C.I. %**	**Protein MW/Da**	**Peptide Count**
Integrase		gi|581947497	73	98.727	40058.5	12
**Calc. Mass**	**Obs. Mass**	**Peptide Seq.**		**Modification**		
916.5462	916.5283	LTTLNNIK				
932.5272	932.5071	RSISINSR				
939.5007	939.5355	RVGHVDEK				
968.4796	968.5488	DHILNDNK				
1006.5163	1006.5793	NNKSSSELK				
1036.5455	1036.5771	TTKALNMNK		Oxidation (M)[7]		
1254.6802	1254.6389	VGHVDEKTTIR				
1346.7175	1346.653	DHILNDNKLHK				
1625.8792	1625.8269	GNPLCNNQIAGVLKK		Carbamidomethyl (C)[5]		
1625.8792	1625.8269	KGNPLCNNQIAGVLK		Carbamidomethyl (C)[6]		
1637.8745	1637.8297	YSSENLLLNKLDTK				
2239.1433	2239.1545	YAVKFYNYPNEYLLTNVK				

Supplementary Table 16. Peptide mass fingerprints of Bcp

Protein Name		Accession No.	Protein Score	Protein Score C.I. %	Protein MW/Da	Peptide Count
AhpC/TSA family protein		gi|49486681	72	98.581	17261.9	13
**Calc. Mass**	**Obs. Mass**	**Peptide Seq.**		**Modification**	**Ion Score**	**C.I. %**
915.4683	915.5018	HQNFIEK				
965.5091	965.5569	GEQFPIFK				
1042.572	1042.6227	AIIYFYPR			14	
1042.6508	1042.6227	VLDVIEKVK				
1065.5939	1065.6254	ETGVYQLKK				
1170.667	1170.7225	KAIIYFYPR				
1170.667	1170.7225	KAIIYFYPR				
1226.6199	1226.6749	SFGKESMGIVR		Oxidation(M)[7]		

Supplementary Table 17. Peptide mass fingerprints of Pgl

**Protein Name**		**Accession No.**	**Protein Score**	**Protein Score C.I. %**	**Protein MW/Da**	**Peptide Count**
6-phosphogluconolactonase		gi|379021683	139	100	38866	12
**Calc. Mass**	**Obs. Mass**	**Peptide Seq.**		**Modification**	**Ion Score**	**C.I. %**
987.5258	987.5399	GHDSIAIFK				
992.4829	992.5269	ITLCDNTR		Carbamidomethyl (C)[4]		
1096.5171	1096.5408	HIEFHDNGK				
1136.5331	1136.5538	FELNENQSR				
1136.5331	1136.5538	FELNENQSR			11	
1163.6056	1163.631	NNEVLYGINK				
1179.6005	1179.6173	YVAVTDLGADR				
1380.7118	1380.7405	IDDNGELHLLNK				
1486.7173	1486.7294	HLTIPENFDGDTK				
1542.7217	1542.7333	AGTGCYVSISEDKR		Carbamidomethyl (C)[5]		
1576.7788	1576.7906	MYELNTHTGEIIR				
1592.7737	1592.7842	MYELNTHTGEIIR		Oxidation (M)[1]		
1606.7972	1606.8064	LSHDQQFLYVSNR				
1606.7972	1606.8064	LSHDQQFLYVSNR			37	
1857.8628	1857.8673	QDHPHAHYINQTPDGK				
1863.9348	1863.9404	LIQELAHDFPTGTHER				

Supplementary Table 18. Peptide mass fingerprints of Hmp

Protein Name		Accession No.	Protein Score	Protein Score C.I. %	Protein MW/Da	Peptide Count
Nitric oxide dioxygenase		gi|581611265	139	100	42992.9	11
**Calc. Mass**	**Obs. Mass**	**Peptide Seq.**		**Modification**	**Ion Score**	**C.I. %**
915.5046	915.4909	NHLTFGVK				
1017.5727	1017.5832	LYTHLKDK				
1029.4307	1029.4399	SLNYDMDR		Oxidation (M)[6]		
1029.4307	1029.4399	SLNYDMDR		Oxidation (M)[6]	2	
1043.5997	1043.5293	KNHLTFGVK				
1161.6051	1161.6019	VHYETFIPR				
1161.6051	1161.6019	VHYETFIPR			42	
1179.6443	1179.6171	FLQSMIEALK				
1195.6392	1195.6249	FLQSMIEALK		Oxidation (M)[5]		
1255.6569	1255.6414	GTEITSIFYPK				
1417.7533	1417.7357	ITNIKQESEDIK				
1512.7944	1512.7756	EKGTEITSIFYPK				
1569.7041	1569.692	DNFNSIASHHDNAK				
1784.8749	1784.863	AHPELLNMFNQTNQK				
1800.8698	1800.8392	AHPELLNMFNQTNQK		Oxidation (M)[8]		
1800.8698	1800.8392	AHPELLNMFNQTNQK		Oxidation (M)[8]	20	

Supplementary Table 19. Peptide mass fingerprints of 6PGDH

Protein Name		Accession No.	Protein Score	Protein Score C.I. %	Protein MW/Da	Peptide Count
6-phosphogluconate dehydrogenase, decarboxylating		gi|487390600	149	100	51899.1	14
**Calc. Mass**	**Obs. Mass**	**Peptide Seq.**		**Modification**	**Ion Score**	**C.I. %**
920.52	920.4958	DFLEKIR				
988.5859	988.5583	ILLMVQAGK		Oxidation (M)[4]		
1028.516	1028.511	GYSVSVFNR				
1028.516	1028.511	GYSVSVFNR			28	
1057.5889	1057.5483	GADILDAIAAK				
1090.5714	1090.5651	LGDLAMIWR		Oxidation (M)[6]		
1102.564	1102.5604	NLAWNIESR				
1108.5997	1108.5938	FISSIKEER				
1108.5997	1108.5938	FISSIKEER			11	
1236.614	1236.6144	TDLMVEESKGK				
1258.5488	1258.5378	DYFGAHTYER				
1258.5488	1258.5378	DYFGAHTYER			40	
1321.6747	1321.6533	NIVTEYQDALR				
1380.7594	1380.7433	AADLPANLIQAQR				
1380.7594	1380.7433	AADLPANLIQAQR			70	
1386.6437	1386.6309	DYFGAHTYERK				
1430.8036	1430.78	TQQIGVIGLAVMGK		Oxidation (M)[12]		
1447.6458	1447.6259	ICSYAQGFAQMR		Carbamidomethyl (C)[2], Oxidation (M)[11]		

Supplementary Table 20. Peptide mass fingerprints of FtnA

**Protein Name**		**Accession No.**	**Protein Score**	**Protein Score C.I. %**	**Protein MW/Da**	**Peptide Count**
Ferritin		gi|582593655	94	99.99	19504.3	6
**Calc. Mass**	**Obs. Mass**	**Peptide Seq.**		**Modification**	**Ion Score**	**C.I. %**
1037.4899	1037.495	ELGARTFDE				
1070.5265	1070.4968	IYNYINDR				
1112.5735	1112.5442	FYNLSEIAR				
1112.5735	1112.5442	FYNLSEIAR			5	
1268.6746	1268.6335	RFYNLSEIAR				
1838.8933	1838.8314	IYNYINDRGAHAEFR				
2027.0081	2026.9498	IGDDSNALYLYEKELGAR				
2027.0081	2026.9498	IGDDSNALYLYEKELGAR			39	

Supplementary Table 21. Peptide mass fingerprints of RplI

Protein Name		Accession No.	Protein Score	Protein Score C.I. %	Protein MW/Da	Peptide Count
50S ribosomal protein L9		gi|446787041	129	100	16629	12
**Calc. Mass**	**Obs. Mass**	**Peptide Seq.**		**Modification**	**Ion Score**	**C.I. %**
1134.6517	1134.5811	VIFTQDVKGK				
1134.6517	1134.5811	VIFTQDVKGK				
1159.6317	1159.6002	LDKEVEGTIR				
1208.6708	1208.613	MKVIFTQDVK				
1208.6708	1208.613	MKVIFTQDVK			21	
1224.6658	1224.5812	MKVIFTQDVK		Oxidation (M)[1]		
1276.6532	1276.6042	NYAVEATPGNLK				
1394.7275	1394.6884	TGEGGKLFGSVNTK				
1404.7482	1404.6825	KNYAVEATPGNLK				
1463.7893	1463.7109	EVPVGYANNFLLK				
1496.7704	1496.7173	EVEGTIRVHTVEQ				
1591.8843	1591.798	EVPVGYANNFLLKK				
1877.0167	1876.8635	GEVKEVPVGYANNFLLK				
2016.076	2015.976	NYAVEATPGNLKQLELQ				

Supplementary Table 22. Peptide mass fingerprints of AhpD

Protein Name		Accession No.	Protein Score	Protein Score C.I. %	Protein MW/Da	Peptide Count
Alkylhydroperoxidase		gi|447069508	220	100	16617.6	8
**Calc. Mass**	**Obs. Mass**	**Peptide Seq.**		**Modification**	**Ion Score**	**C.I. %**
920.52	920.5982	KFEIIDR				
943.5571	943.6491	LNIISDRL				
943.5571	943.6491	LNIISDRL			40	
978.5255	978.6226	IDEVLSFR				
978.5255	978.6226	IDEVLSFR			52	
1033.6041	1033.7073	FEIIDRLK				
1033.6041	1033.7073	FEIIDRLK			17	
1245.6111	1245.7292	HLDLFTDQEK			50	
1275.6978	1275.8059	ELKELGVTQMK				
1553.6935	1553.8864	QNGELMSVMSQGEK		Oxidation (M)[6]		
1681.7885	1681.952	KQNGELMSVMSQGEK		Oxidation (M)[7]		

Supplementary Table 23. Peptide mass fingerprints of AhpD family core domain protein

Protein Name	Accession No.	Protein Score	Protein Score C.I. %	Protein MW/Da	Peptide Count
alkylhydroperoxidase AhpD family core domain protein	gi|377718914	67	94.989	14804.7	4
**Calc. Mass**	**Obs. Mass**	**Peptide Seq.**	**Modification**	**Ion Score**	**C.I. %**
943.5571	943.6372	LNIISDRL			
943.5571	943.6372	LNIISDRL		12	
978.5255	978.6098	IDEVLSFR			
978.5255	978.6098	IDEVLSFR			
1033.6041	1033.6744	FEIIDRLK			

Supplementary Table 24. Peptide mass fingerprints of Tnp

Protein Name		Accession No.	Protein Score	Protein Score C.I. %	Protein MW/Da	Peptide Count
Transposase		gi|537468406	163	100	20154.4	7
**Calc. Mass**	**Obs. Mass**	**Peptide Seq.**		**Modification**	**Ion Score**	**C.I. %**
920.52	920.5982	KFEIIDR				
978.5255	978.6226	IDEVLSFR				
978.5255	978.6226	IDEVLSFR			52	
1033.6041	1033.7073	FEIIDRLK				
1033.6041	1033.7073	FEIIDRLK			17	
1245.6111	1245.7292	HLDLFTDQEK			50	
1275.6978	1275.8059	ELKELGVTQMK				
1553.6935	1553.8864	QNGELMSVMSQGEK		Oxidation (M)[6]		
1681.7885	1681.952	KQNGELMSVMSQGEK		Oxidation (M)[7]		

Supplementary Table 25. Peptide mass fingerprints of TelA

**Protein Name**	**Accession No.**	**Protein Score**	**Protein Score C.I. %**	**Protein MW/Da**	**Peptide Count**
tellurite resistance protein TelA	gi|657833828	203	100	43337.5	12
**Calc. Mass**	**Obs. Mass**	**Peptide Seq.**	**Modification**	**Ion Score**	**C.I. %**
1007.552	1007.6208	IYDLQLSR			
1007.552	1007.6208	IYDLQLSR		41	
1010.5339	1010.6471	MTENKIFK			
1033.5525	1033.62	ELSNTTIQK			
1052.5371	1052.6088	SSINEIFSR			
1052.5371	1052.6088	SSINEIFSR		27	
1120.5416	1120.624	MQSVSAQVDR			
1129.6576	1129.7324	GIVDLDTLKR			
1238.7216	1238.8118	QIALQTAPQIR			
1238.7216	1238.8118	QIALQTAPQIR		46	
1251.6692	1251.7627	AKSSINEIFSR			
1251.6692	1251.7627	AKSSINEIFSR		6	
1261.6755	1261.7716	NQMAIALTLMR			
1277.6704	1277.7465	NQMAIALTLMR	Oxidation (M)[3]		
1293.6654	1293.7417	NQMAIALTLMR	Oxidation (M)[3,10]		
1358.7097	1358.7704	MIQNVNQALAEK			
1374.7046	1374.8304	MIQNVNQALAEK	Oxidation (M)[1]		
1375.6448	1375.7542	QNAIETATENER			
1943.0015	1943.1295	SVNPNELNTDKPSMLKR			
1958.9965	1959.1123	SVNPNELNTDKPSMLKR	Oxidation (M)[14]		

Supplementary Table 26. Peptide mass fingerprints of RplK

Protein Name		Accession No.	Protein Score	Protein Score C.I. %	Protein MW/Da	Peptide Count
50S ribosomal protein L11		gi|613363411	67	94.752	14907.8	4
**Calc. Mass**	**Obs. Mass**	**Peptide Seq.**		**Modification**	**Ion Score**	**C.I. %**
935.5924	935.6311	TPPAPVLLK			46	
1063.6874	1063.8099	TPPAPVLLKK				
1159.6066	1159.7472	DQVREIANSK				
1564.673	1564.807	MQDLNAADEEAAMR				
1580.668	1580.7905	MQDLNAADEEAAMR		Oxidation (M)[1]		

Supplementary Table 27. Peptide mass fingerprints of Dps

Protein Name		Accession No.	Protein Score	Protein Score C.I. %	Protein MW/Da	Peptide Count
general stress protein		gi|584762748	328	100	18033.4	6
**Calc. Mass**	**Obs. Mass**	**Peptide Seq.**		**Modification**	**Ion Score**	**C.I. %**
956.5047	956.5629	ETTDLLHK				
1273.6747	1273.7355	LTEDIQGKVDR				
1325.7173	1325.77	ETTDLLHKVNR				
1325.7173	1325.77	ETTDLLHKVNR			91	
1472.7704	1472.8356	TLDGVEGQVQGITR				
1472.7704	1472.8356	TLDGVEGQVQGITR			81	
1547.766	1547.8336	GIGDSVQTLNSSVDR				
1620.7361	1620.8038	ANNVATDANHSYTSR				
1620.7361	1620.8038	ANNVATDANHSYTSR			107	

Supplementary Table 28. Peptide mass fingerprints of RpoB

Protein Name		Accession No.	Protein Score	Protein Score C.I. %	Protein MW/Da	Peptide Count
DNA-directed RNA polymerase subunit beta'		gi|578916640	326	100	135855.6	22
**Calc. Mass**	**Obs. Mass**	**Peptide Seq.**		**Modification**	**Ion Score**	**C.I. %**
1000.5608	1000.5406	SLSHMLKGK				
1087.6041	1087.675	LIPAGTGMRR		Oxidation (M)[8]		
1134.6266	1134.7101	RLEVVESFR				
1348.7809	1348.8784	GRPVTGPGNRPLK				
1382.8115	1382.7567	RLVDVAQDVIVR				
1385.7073	1385.8066	AYANGFVHLHTR				
1392.7634	1392.8616	FLGNIIAEVFNR				
1394.7638	1394.7507	ELVQHEIATNIK				
1394.7638	1394.7507	ELVQHEIATNIK				
1425.7042	1425.8032	ITDAGIEQMYIR		Oxidation (M)[9]		
1470.7336	1470.8368	FATSDLNDLYRR				
1487.7812	1487.8817	LLRDELESATGQR				
1529.8323	1529.9415	LGIQAFEPTLVEGR				
1529.8323	1529.9415	LGIQAFEPTLVEGR			51	
1553.7992	1553.9048	KITDAGIEQMYIR		Oxidation (M)[10]		
1593.021	1592.921	KRPATAKPVLLGITK				
1652.9343	1652.9192	EHPVLLNRAPTLHR				
1716.9642	1716.9843	KPETINYRTLKPEK				
1736.0065	1736.1243	LLDLGAPGIIVQNEKR				
1792.9229	1793.0481	EGLTVLEYFISTHGAR				
1813.9192	1814.0454	TFHTGGVAGSDITQGLPR				
1813.9192	1814.0454	TFHTGGVAGSDITQGLPR			93	
1881.9818	1882.109	LLPGSLVDIHNFTDANR				
1881.9818	1882.109	LLPGSLVDIHNFTDANR			77	
2074.9927	2075.1321	ASLETESFLSAASFQETT				
2248.0994	2248.2576	IGVHASSFNNPTFTEEQN				

Supplementary Table 29. Peptide mass fingerprints of PflB

Protein Name		Accession No.	Protein Score	Protein Score C.I. %	Protein MW/Da	Peptide Count
formate acetyltransferase		gi|446817403	111	100	85264	16
**Calc. Mass**	**Obs. Mass**	**Peptide Seq.**		**Modification**	**Ion Score**	**C.I. %**
944.5411	944.5844	ELKELGQK				
1008.4931	1008.5687	SMQPFGGIR		Oxidation (M)[2]		
1322.6699	1322.7661	LREELSEQYR			36	
1322.6708	1322.7661	SMQPFGGIRMAK				
1417.7798	1417.8839	KAGVITGLPDAYGR				
1426.736	1426.8389	IEMALHDTEIVR				
1442.7308	1442.8295	IEMALHDTEIVR		Oxidation (M)[3]		
1522.7397	1522.8514	KTHNQGVFDAYSR				
1543.754	1543.8645	YGFDISRPAENFK				
1636.8013	1636.9218	KAGEPFAPGANPMHGR				
1652.7963	1652.9099	KAGEPFAPGANPMHGR		Oxidation (M)[13]		
1669.8618	1669.9769	RVALYGVDFLMEEK				
1675.8473	1675.9663	LWEQVMQLSKEER				
1691.8422	1691.9578	LWEQVMQLSKEER		Oxidation (M)[6]		
1860.0226	1860.1543	DLETIVGVQTEKPFKR				
2058.8567	2059.0081	MHDFNTMSTEMSEDVIR		Oxidation (M)[1]		
2077.9785	2078.1162	YGNNDDRVDDIAVDLVER				
2285.1118	2285.2683	ETLIDAMEHPEEYPQLTIR				
2301.1067	2301.2664	ETLIDAMEHPEEYPQLTIR		Oxidation (M)[7]		
2892.4778	2892.6562	YAQVKPIRNEEGLVVDFE IEGDFPK				

Supplementary Table 30. Peptide mass fingerprints of CodY

Protein Name	Accession No.	Protein Score	Protein Score C.I. %	Protein MW/Da	Peptide Count
transcriptional repressor CodY	gi|686242013	188	100	28738.3	8

Calc. Mass	Obs. Mass	Peptide Seq.	Modification	Ion Score	C.I. %
935.5342	935.467	MSLLSKTR			
951.5291	951.5109	MSLLSKTR	Oxidation (M)[1]		
986.5742	986.5186	VADRVGITR			
986.5742	986.5186	VADRVGITR		3	
1014.5731	1014.4998	HKGIAVDFK			
1031.5554	1031.5055	IIQMLEER			
1031.5554	1031.5055	IIQMLEER		14	
1047.5503	1047.4869	IIQMLEER	Oxidation (M)[4]		
1131.543	1131.4938	HIPSEYTER			
1131.543	1131.4938	HIPSEYTER		32	
1188.6583	1188.605	KLESAGVIESR			
1188.6583	1188.605	KLESAGVIESR		56	
1215.7056	1215.6239	TRELNTLLQK			
1260.6947	1260.6351	TTIFPILGGGER			
1260.6947	1260.6351	TTIFPILGGGER		31	

Supplementary Table 31. Peptide mass fingerprints of RpoA

Protein Name		Accession No.	Protein Score	Protein Score C.I. %	Protein MW/Da	Peptide Count
DNA-directed RNA polymerase subunit alpha (partial)		gi|15927804	249	100	32433.160	12
**Calc. Mass**	**Obs. Mass**	**Peptide Seq.**		**Modification**	**Ion Score**	**C.I. %**
985.5676	985.6354	LEDLGLGLR				
1031.5554	1031.6343	MIEIEKPR		Oxidation (M)[1]		
1095.543	1095.6351	VNYTVENTR				
1138.5852	1138.6724	GYGTTLGNSLR				
1168.73	1168.8127	ILLSSLPGAAVK				
1247.6366	1247.7397	IETIEISEDAK				
1276.726	1276.822	YKLEDLGLGLR				
1294.6863	1294.7892	GYGTTLGNSLRR				
1320.7311	1320.8237	FGKFVVEPLER				
1324.8312	1324.9213	RILLSSLPGAAVK				
1648.8462	1648.9722	VLEMSIEELDLSVR		Oxidation (M)[4]		
1709.8593	1709.9854	IYSEEDKTLEIDVR				

Supplementary Table 32. Peptide mass fingerprints of Ldh1

Protein Name		Accession No.	Protein Score	Protein Score C.I. %	Protein MW/Da	Peptide Count
lactate dehydrogenase 1		gi|446954621	111	100	34648	9
**Calc. Mass**	**Obs. Mass**	**Peptide Seq.**		**Modification**	**Ion Score**	**C.I. %**
1032.5507	1032.6008	VRGDVMDLK				
1107.5681	1107.6143	DAAYDIIQAK				
1156.6321	1156.6443	SLLEQRPEGK				
1156.6321	1156.6443	SLLEQRPEGK				
1188.6583	1188.7162	VIGSGTILDSAR				
1229.6273	1229.6925	HATPYSPTTVR				
1229.6273	1229.6925	HATPYSPTTVR			12	
1329.662	1329.7343	GATYYGVAMGLAR				
1330.7366	1330.785	LLLSEAFDVAPR				
1332.7271	1332.7915	AQIEQIFVQTR				
1332.7271	1332.7915	AQIEQIFVQTR			39	
1456.7908	1456.8459	HATPYSPTTVRVK				

Supplementary Table 33. Peptide mass fingerprints of TrxA

Protein Name	Accession No.	Protein Score	Protein Score C.I. %	Protein MW/Da	Peptide Count
Thioredoxin	gi|446104989	100	99.997	21958.1	7

Calc. Mass	Obs. Mass	Peptide Seq.	Modification	Ion Score	C.I. %
1012.5131	1012.575	IKEMNYSK			
1015.5095	1015.5281	TNLETYFK			
1068.5797	1068.5985	ETHLVISNR			
1131.5542	1131.5496	VFHRDDNTK			
1280.6958	1280.7136	HISEALNLEVR			
1280.6958	1280.7136	HISEALNLEVR		50	
1405.7434	1405.7588	ANEVQKFVTDVR			
1505.8322	1505.817	AVYNSILNKLENK			

Supplementary Table 34. Peptide mass fingerprints of TrxB

Protein Name		Accession No.	Protein Score	Protein Score C.I. %	Protein MW/Da	Peptide Count
thioredoxin reductase		gi|446057106	204	100	33723.1	11
**Calc. Mass**	**Obs. Mass**	**Peptide Seq.**		**Modification**	**Ion Score**	**C.I. %**
992.5623	992.55	VGSVTLTSTK				
1054.5051	1054.5618	SVEDKGEYK				
1135.6357	1135.6187	AVIIATGAEYK				
1185.674	1185.6715	FADKVTIVHR				
1190.6562	1190.6573	ANLKTVMIER		Oxidation (M)[7]		
1211.6379	1211.6388	IGVPGEQELGGR				
1211.6379	1211.6388	IGVPGEQELGGR			76	
1260.626	1260.618	FGAVYQYGDIK				
1263.7307	1263.713	AVIIATGAEYKK				
1263.7307	1263.713	AVIIATGAEYKK			49	
1333.7474	1333.73	VINFGNKELTAK				
1339.7329	1339.7365	KIGVPGEQELGGR				
1388.7209	1388.71	KFGAVYQYGDIK				

Supplementary Table 35. Peptide mass fingerprints of Tuf

Protein Name		Accession No.	Protein Score	Protein Score C.I. %	Protein MW/Da	Peptide Count
elongation factor Tu		gi|446963312	302	100	43133.6	14
**Calc. Mass**	**Obs. Mass**	**Peptide Seq.**		**Modification**	**Ion Score**	**C.I. %**
1045.575	1045.577	GTVATGRVER				
1140.5718	1140.5779	TTVTGVEMFR				
1140.5718	1140.5779	TTVTGVEMFR			56	
1156.5668	1156.566	TTVTGVEMFR		Oxidation (M)[8]		
1252.5692	1252.5851	ALEGDAQYEEK				
1268.6667	1268.67	TTVTGVEMFRK				
1284.6617	1284.6599	TTVTGVEMFRK		Oxidation (M)[8]		
1422.7224	1422.7305	AEVYVLSKDEGGR				
1703.8962	1703.8937	LLDYAEAGDNIGALLR				
1752.928	1752.9235	GQVLAAPGSITPHTEFK				
1768.7861	1768.7917	HYAHVDCPGHADYVK		Carbamidomethyl (C)[7]		
1799.9148	1799.9188	SKEHANIGTIGHVDHGK				
1831.9912	1832.0006	KLLDYAEAGDNIGALLR				
1875.9559	1875.9592	GITINTSHIEYQTDKR				
1875.9559	1875.9592	GITINTSHIEYQTDKR			103	
1990.0201	1990.0538	ILELMEAVDTYIPTPER				
2006.9661	2006.973	HTPFFSNYRPQFYFR				
2006.9661	2006.973	HTPFFSNYRPQFYFR			28	
2368.0356	2368.0442	NGDSVAQSYDMIDNAPE				

Supplementary Table 36. Peptide mass fingerprints of Eno1

Protein Name		Accession No.	Protein Score	Protein Score C.I. %	Protein MW/Da	Peptide Count
alpha-enolase		gi|555636526	276	100	47209.8	15
**Calc. Mass**	**Obs. Mass**	**Peptide Seq.**		**Modification**	**Ion Score**	**C.I. %**
1030.5065	1030.5212	FEGEHGAKR				
1045.5637	1045.577	QLTDRIGDK				
1060.5535	1060.5719	AGYTAVVSHR				
1118.6681	1118.6361	IAKYNQLLR				
1168.6031	1168.5583	MSIITDVYAR				
1259.6995	1259.6813	AAADLLGQPLYK				
1422.708	1422.7305	MMIQLDGTSNKGK				
1424.8584	1424.8145	LGANAILGVSIAVAR				
1447.7064	1447.7	GLETAVGDEGGFAPK				
1603.8075	1603.8119	RGLETAVGDEGGFAPK				
1603.8075	1603.8119	RGLETAVGDEGGFAPK			76	
1720.8752	1720.8325	SAAEQVDYLEQLINK				
1770.8796	1770.8384	IEDELYETAKFDGIK				
1822.9294	1822.9407	ALVPSGASTGEHEAVELR				
1822.9294	1822.9407	ALVPSGASTGEHEAVELR			84	
1861.9291	1861.9392	GNPTVEVEVLTESGAFGR				
2238.0996	2238.1111	ALVPSGASTGEHEAVEL RDGDK				

Supplementary Table 37. Peptide mass fingerprints of Mqo2

Protein Name		Accession No.	Protein Score	Protein Score C.I. %	Protein MW/Da	Peptide Count
malate:quinone oxidoreductase		gi|447052793	202	100	56138.6	14
**Calc. Mass**	**Obs. Mass**	**Peptide Seq.**		**Modification**	**Ion Score**	**C.I. %**
1046.589	1046.5509	KWIPLMMK				
1131.6166	1131.6047	WIPLMMKGR				
1131.6166	1131.6047	WIPLMMKGR				
1231.6028	1231.6357	YSFDQVIMTK				
1345.653	1345.6576	GREDNPGIMAASK				
1360.7625	1360.7427	TLLFGPFANVGPK				
1386.6971	1386.688	LDRPAIESSNER				
1480.7179	1480.7092	NEDWQLYTAGKR				
1480.7179	1480.7092	NEDWQLYTAGKR			64	
1509.7373	1509.741	NFPEYKTEWAPK				
1565.7443	1565.7474	IDEGTDVNFGELTR				
1584.8533	1584.8403	EFINPLPHISYVR				
1584.8533	1584.8403	EFINPLPHISYVR			46	
1693.8392	1693.8334	IDEGTDVNFGELTRK				
1699.8075	1699.8174	EIEPDWNIHVYER				
1744.8688	1744.8527	EPPGTPPMTVPHLDTR				
2192.1169	2192.1211	VYGKEPPGTPPMTVPHLD TR				

Supplementary Table 38. Peptide mass fingerprints of UPF0356 protein

Protein Name		Accession No.	Protein Score	Protein Score C.I. %	Protein MW/Da	Peptide Count
UPF0356 protein		gi|487385298	104	99.999	8718.3	5
**Calc. Mass**	**Obs. Mass**	**Peptide Seq.**		**Modification**	**Ion Score**	**C.I. %**
963.4795	963.5377	VFYQHNR				
963.4795	963.5377	VFYQHNR			22	
1125.5939	1125.6525	NFNIEFITK				
1174.5739	1174.6459	LEGAHLDYEK				
1416.6754	1416.7534	ENSEHFNVEIAK				
1674.8711	1674.9574	VFYQHNRDEVIVR				
1674.8711	1674.9574	VFYQHNRDEVIVR			33	

Supplementary Table 39. Peptide mass fingerprints of cysteine synthase (CysK)

Protein Name		Accession No.	Protein Score	Protein Score C.I. %	Protein MW/Da	Peptide Count
cysteine synthase		gi|445979740	127	100	33040.2	5
**Calc. Mass**	**Obs. Mass**	**Peptide Seq.**		**Modification**	**Ion Score**	**C.I. %**
904.5172	904.5574	IALAMIEK		Oxidation (M)[5]		
1244.7031	1244.7776	IALAMIEKAER				
1244.7031	1244.7776	IALAMIEKAER			18	
1271.6954	1271.7754	TVVTVLPSNGER				
1271.6954	1271.7754	TVVTVLPSNGER			27	
1462.7285	1462.8185	LEYQNPGGSVKDR				
1462.7285	1462.8185	LEYQNPGGSVKDR			29	
1526.6979	1526.7982	AVFTMPETMSQER				
1542.6927	1542.7781	AVFTMPETMSQER		Oxidation (M)[5]		

Supplementary Table 40. Peptide mass fingerprints of CcpA

**Protein Name**		**Accession No.**	**Protein Score**	**Protein Score C.I. %**	**Protein MW/Da**	**Peptide Count**
catabolite control protein A		gi|686335442	163	100	36137.7	8
**Calc. Mass**	**Obs. Mass**	**Peptide Seq.**		**Modification**	**Ion Score**	**C.I. %**
943.5571	943.6024	NKVNEVIK				
971.5269	971.5671	VVNGNQNVK				
1031.5619	1031.6041	EITGELIEK				
1037.5626	1037.6086	TVTIYDVAR				
1131.5793	1131.632	SFALVGGEHSK				
1131.5793	1131.632	SFALVGGEHSK			43	
1173.6487	1173.6981	LNYRPNAVAR				
1173.6487	1173.6981	LNYRPNAVAR			56	
1259.6743	1259.7241	SFALVGGEHSKK				
1329.7499	1329.7999	RLNYRPNAVAR				
1329.7499	1329.7999	RLNYRPNAVAR			17	

Supplementary Table 41. Peptide mass fingerprints of PfkA

Protein Name	Accession No.	Protein Score	Protein Score C.I. %	Protein MW/Da	Peptide Count
6-phosphofructokinase	gi|446640221	124	100	35131.1	9

Calc. Mass	Obs. Mass	Peptide Seq.	Modification	Ion Score	C.I. %
942.5731	942.6073	VAIENLRK			
970.5792	970.6055	RVAIENLR			
971.4945	971.5359	GGTFLYSAR			
971.4945	971.5359	GGTFLYSAR		40	
994.5792	994.6194	VSVLGHVQR			
994.5792	994.6194	VSVLGHVQR		26	
1037.5449	1037.5955	TFIIEAMGR			
1053.5398	1053.5775	TFIIEAMGR	Oxidation (M)[7]		
1287.6903	1287.7396	LELGSVGDTIQR			
1350.6648	1350.7208	ELSQYINVDNR			
1491.7803	1491.8348	GIEGLVVIGGDGSYR			
1715.8745	1715.9357	IAVLTSGGDSPGMNAAVR			

Supplementary Table 42. Peptide mass fingerprints of PepP

Protein Name	Accession No.	Protein Score	Protein Score C.I. %	Protein MW/Da	Peptide Count
peptidase M24	gi|686441981	120	100	39523.4	8

Calc. Mass	Obs. Mass	Peptide Seq.	Modification	Ion Score	C.I. %
996.5697	996.6105	SRITQVHR			
1055.5521	1055.595	QYLITDFR			
1074.5466	1074.584	TFAIGEPDPK			
1127.6102	1127.6875	MSRITQVHR			
1217.6273	1217.6786	QAPNYEIINR			
1217.6273	1217.6786	QAPNYEIINR		50	
1298.674	1298.7239	DKQYLITDFR			
1345.7223	1345.775	QAPNYEIINRK			
1345.7223	1345.775	QAPNYEIINRK		28	
1971.9917	1972.0551	AINEIRPGMTGAEADAISR			

Supplementary Table 43. Peptide mass fingerprints of TrpS

Protein Name		Accession No.	Protein Score	Protein Score C.I. %	Protein MW/Da	Peptide Count
tryptophanyl-tRNA synthetase		gi|570299737	148	100	36736.9	9
**Calc. Mass**	**Obs. Mass**	**Peptide Seq.**		**Modification**	**Ion Score**	**C.I. %**
902.389	902.4238	YEGEGYGK				
1044.5321	1044.5764	LDDILDQGR				
1046.5299	1046.576	VMSLQDPTR				
1062.5249	1062.5463	VMSLQDPTR		Oxidation (M)[2]		
1110.583	1110.6449	AFLVEFQEK				
1119.6409	1119.6672	FKGDLAEIVK				
1120.5858	1120.6188	NLVDRFNSR				
1120.5858	1120.6188	NLVDRFNSR			22	
1174.6249	1174.66	VMSLQDPTRK				
1190.6199	1190.6455	VMSLQDPTRK		Oxidation (M)[2]		
1345.7474	1345.7886	YNDVLVKPEIR				
1345.7474	1345.7886	YNDVLVKPEIR			71	
1523.77	1523.8152	SAVTDSDGIIKFDR				

Supplementary Table 44. Peptide mass fingerprints of universal stress protein UspA

Protein Name		Accession No.	Protein Score	Protein Score C.I. %	Protein MW/Da	Peptide Count
universal stress protein UspA		gi|446556828	381	100	19150.9	8
**Calc. Mass**	**Obs. Mass**	**Peptide Seq.**		**Modification**	**Ion Score**	**C.I. %**
1129.6576	1129.6973	LTIVNVIDSR				
1129.6576	1129.6973	LTIVNVIDSR			68	
1165.6147	1165.6564	HAPCDVLVVR		Carbamidomethyl (C)[4]		
1165.6147	1165.6564	HAPCDVLVVR		Carbamidomethyl (C)[4]	62	
1292.7209	1292.7694	FIVGSVSESIVR				
1292.7209	1292.7694	FIVGSVSESIVR			100	
1320.6583	1320.676	HFAEELLNGYK				
1377.7009	1377.7531	DVETRLEFGSPK				
1377.7009	1377.7531	DVETRLEFGSPK			85	
1488.7653	1488.8448	EVATNAGVKDVETR				
1535.7853	1535.7853	SKHFAEELLNGYK				
2044.0347	2044.119	TEELPADFQPQVATTQLR				

Supplementary Table 45. Peptide mass fingerprints of PdhB

Protein Name		Accession No.	Protein Score	Protein Score C.I. %	Protein MW/Da	Peptide Count
Pyruvate dehydrogenase E1 component beta subunit		gi|872535879	214	100	113	100
**Calc. Mass**	**Obs. Mass**	**Peptide Seq.**		**Modification**	**Ion Score**	**C.I. %**
999.5582	999.562	AVVVQEAQR				
999.5582	999.562	AVVVQEAQR			52	
1139.6783	1139.6853	AILSLEAPIGR				
1139.6783	1139.6853	AILSLEAPIGR			61	
1145.6201	1145.5912	VVIPSGPYDAK				
1217.6848	1217.6536	SGSTKTAPVTIR				
1265.6372	1265.6453	DGYSVEVIDLR				
1431.6937	1431.7028	SNDPVVYLEHMK				
1447.6886	1447.6832	SNDPVVYLEHMK		Oxidation (M)[11]		
1456.7755	1456.7839	QAGVGAAVVAELSER				
1507.7388	1507.7494	VTEGLQKEFGEDR				
1863.9421	1863.955	SNDPVVYLEHMKLYR				
1879.9371	1879.9292	SNDPVVYLEHMKLYR		Oxidation (M)[11]		
1985.1431	1985.1481	VVIPSGPYDAKGLLISSIR				
2028.0973	2028.0995	TVQPIDVDTIVASVEKTGR				
2036.0182	2036.0205	AAEELEKDGYSVEVIDLR				
2040.9761	2040.9771	SFREEVPEEEYTIDIGK				
2841.4531	2841.4263	SPFGGGVHTPELHADNL EGILAQSPGLK				

Supplementary Table 46. Peptide mass fingerprints of RpsB

**Protein Name**	**Accession No.**	**Protein Score**	**Protein Score C.I. %**	**Protein MW/Da**	**Peptide Count**
30S ribosomal protein S2	gi|686127623	170	100	29143.2	9
**Calc. Mass**	**Obs. Mass**	**Peptide Seq.**	**Modification**	**Ion Score**	**C.I. %**
936.5149	936.5319	EYDRLIK			
956.52	956.5631	KYIFTER			
1036.5609	1036.6238	FLGGIRDMK			
1052.5558	1052.627	FLGGIRDMK	Oxidation (M)[8]		
1096.5535	1096.6084	AGQFYINQR			
1096.5535	1096.6084	AGQFYINQR		57	
1226.6416	1226.688	KVDEAYNFLK			
1361.6655	1361.7451	QAQESVKSEAER			
1592.8292	1592.9141	QLLEAGVHFGHQTR			
1592.8292	1592.9141	QLLEAGVHFGHQTR		67	
1731.9099	1731.9656	DMKSIPQALFVVDPR	Oxidation (M)[2]		
1748.9304	1749.0072	QLLEAGVHFGHQTRR			

Supplementary Table 47. Peptide mass fingerprints of Fib

Protein Name	Accession No.	Protein Score	Protein Score C.I. %	Protein MW/Da	Peptide Count
fibrinogen-binding protein	gi|757461200	206	100	12867	7

Calc. Mass	Obs. Mass	Peptide Seq.	Modification	Ion Score	C.I. %
1206.5426	1206.5693	YDQYQTNFK			
1243.614	1243.6954	MNYEKNFIGK			
1334.6376	1334.6149	YDQYQTNFKK			
1334.6376	1334.6149	KYDQYQTNFK		61	
1447.8519	1447.8176	QIDLVLKYNTLK			
1585.8121	1585.7896	AVNLIHFQHSYEK			
1585.8121	1585.7896	AVNLIHFQHSYEK		88	
1713.9071	1713.886	AVNLIHFQHSYEKK			

Supplementary Table 48. Peptide mass fingerprints of electron transfer DM13

Protein Name	Accession No.	Protein Score	Protein Score C.I. %	Protein MW/Da	Peptide Count
electron transfer DM13	gi|447016303	113	100	16191.3	7

Calc. Mass	Obs. Mass	Peptide Seq.	Modification	Ion Score	C.I. %
977.5051	977.4775	HLTGTFSSK			
1027.6047	1027.5767	KAHVIFGGAK			
1099.4977	1099.531	EIAMVDYDK	Oxidation (M)[4]		
1168.6249	1168.5819	GPDLYVYLTK			
1304.6692	1304.6862	NSETVEGKAEIK			
1356.6351	1356.5953	EIAMVDYDKEK	Oxidation (M)[4]		
1470.7839	1470.7528	SSKGPDLYVYLTK			
1470.7839	1470.7528	SSKGPDLYVYLTK		65	

Supplementary Table 49. Peptide mass fingerprints of RplF

Protein Name		Accession No.	Protein Score	Protein Score C.I. %	Protein MW/Da	Peptide Count
50S ribosomal protein L6		gi|686128415	197	100	19759.4	10
**Calc. Mass**	**Obs. Mass**	**Peptide Seq.**		**Modification**	**Ion Score**	**C.I. %**
914.4366	914.4141	YQGEYVR				
914.4366	914.4141	YQGEYVR			33	
1070.5378	1070.5398	YQGEYVRR				
1072.5786	1072.571	SVRPPEPYK				
1104.6412	1104.6301	VLELVGVGYR				
1104.6412	1104.6301	VLELVGVGYR			56	
1157.6273	1157.6151	EQVGALASNIR				
1240.6433	1240.6505	GIRYQGEYVR				
1257.6951	1257.6808	SVRPPEPYKGK				
1257.6951	1257.6808	SVRPPEPYKGK			25	
1696.9269	1696.8599	DLILNVGYSHPVEIK				
1736.9	1736.8595	ALLNNMVQGVSQGYVK		Oxidation (M)[6]		
1770.9708	1770.9321	VEGISKEQVGALASNIR				

Supplementary Table 50. Peptide mass fingerprints of RpsC

**Protein Name**		**Accession No.**	**Protein Score**	**Protein Score C.I. %**	**Protein MW/Da**	**Peptide Count**
30S ribosomal protein S3		gi|686391303	224	100	24115.1	10
**Calc. Mass**	**Obs. Mass**	**Peptide Seq.**		**Modification**	**Ion Score**	**C.I. %**
988.5422	988.5483	GGSEIEKLR				
988.5422	988.5483	GGSEIEKLR			39	
1095.6633	1095.6656	GQKINPIGLR				
1186.658	1186.6644	VGIIRDWEAK				
1255.6277	1255.6315	EASVSHVEIER				
1317.6686	1317.7201	DFTSLLHEDLK				
1460.826	1460.8163	VWIYRGEVLPTK				
1664.9517	1664.9352	INIAIHTGKPGMVIGK		Oxidation (M)[12]		
1700.8602	1700.8696	AEQYSEGTVPLHTLR				
1740.7711	1740.7695	ADIDYAHAEADTTYGK				
2115.0718	2115.0867	FIDNELKEASVSHVEIER				
2115.0718	2115.0867	FIDNELKEASVSHVEIER			109	

Supplementary Table 51. Peptide mass fingerprints of AtpA

Protein Name		Accession No.	Protein Score	Protein Score C.I. %	Protein MW/Da	Peptide Count
ATP synthase subunit alpha		gi|446897627	190	100	54625.4	11
**Calc. Mass**	**Obs. Mass**	**Peptide Seq.**		**Modification**	**Ion Score**	**C.I. %**
1001.5625	1001.5557	AEEISALLR				
1024.615	1024.6156	AIDALVPIGR				
1024.615	1024.6156	AIDALVPIGR			54	
1107.5649	1107.5441	VGGSAQMKAMK				
1186.6315	1186.6284	SVDEPLQTGIK				
1229.6848	1229.6873	ELIIGDRQTGK				
1313.7787	1313.7784	AIKAEEISALLR				
1314.7264	1314.7686	KSVDEPLQTGIK				
1434.8064	1434.7562	VAGTLRLDLASYR				
1441.772	1441.7573	IMEVPVGEELIGR				
1457.7668	1457.7412	IMEVPVGEELIGR		Oxidation (M)[2]		
1475.774	1475.7639	GYLDDIPVVDITR				
1553.7384	1553.7305	EAYPGDVFYLHSR				
1553.7384	1553.7305	EAYPGDVFYLHSR			64	

Supplementary Table 52. Peptide mass fingerprints of protein map

Protein Name		Accession No.	Protein Score	Protein Score C.I. %	Protein MW/Da	Peptide Count
protein map		gi|375369277	245	100	77039.6	22
**Calc. Mass**	**Obs. Mass**	**Peptide Seq.**		**Modification**	**Ion Score**	**C.I. %**
901.5101	901.5001	GIGERELK				
909.504	909.4937	VGYKDISK				
939.4755	939.4698	HDRGIGER				
966.5043	966.4968	ATYTVHFK				
971.5996	971.5908	RILQLNSK				
1054.5244	1054.5205	AYYTVYFK				
1094.5994	1094.5983	KATYTVHFK				
1115.642	1115.6281	AININVDTKK				
1130.5953	1130.5959	NYTANLVHAK				
1182.6194	1182.6069	KAYYTVYFK			43	
1182.6194	1182.6069	KAYYTVYFK				
1206.6729	1206.6658	GISDLDLKFAK				
1216.6896	1216.6932	GITDVDLRLSK			28	
1216.6896	1216.6932	GITDVDLRLSK				
1232.6481	1232.6348	SERGISDLDLK				
1246.6387	1246.6442	SDRGISDIDLR				
1339.6893	1339.6772	SDIFTPNLFSAK				
1366.7114	1366.74	ATYTVHFKNGTK				
1472.7856	1472.7731	NYTANLVHAKDVK				
1494.7184	1494.7312	NQNISYKDLEDR			42	
1494.7184	1494.7312	NQNISYKDLEDR				
1595.8275	1595.819	SGIYTANLINSSDIK				
1608.8479	1608.8334	AGIYTADLINTSEIK				
1673.8817	1673.8821	SVLESNRGITDVDLR				
